# Vaginal Microbiome Metagenome Inference Accuracy: Differential Measurement Error according to Community Composition

**DOI:** 10.1128/msystems.01003-22

**Published:** 2023-03-28

**Authors:** Kayla A. Carter, Anthony A. Fodor, Jennifer E. Balkus, Angela Zhang, Myrna G. Serrano, Gregory A. Buck, Stephanie M. Engel, Michael C. Wu, Shan Sun

**Affiliations:** a Department of Epidemiology, University of Washington, Seattle, Washington, USA; b Department of Bioinformatics and Genomics, University of North Carolina at Charlotte, Charlotte, North Carolina, USA; c Vaccine and Infectious Disease Division, Fred Hutchinson Cancer Research Center, Seattle, Washington, USA; d Department of Biostatistics, University of Washington, Seattle, Washington, USA; e Department of Microbiology and Immunology, School of Medicine, Virginia Commonwealth University, Richmond, Virginia, USA; f Center for Microbiome Engineering and Data Analysis, Virginia Commonwealth University, Richmond, Virginia, USA; g Department of Computer Science, College of Engineering, Virginia Commonwealth University, Richmond, Virginia, USA; h Department of Epidemiology, University of North Carolina at Chapel Hill, Chapel Hill, North Carolina, USA; i Public Health Sciences Division, Fred Hutchinson Cancer Research Center, Seattle, Washington, USA; University of California San Diego; University of California San Diego

**Keywords:** *Lactobacillus crispatus*, *Lactobacillus iners*, measurement error, metagenome inference, vaginal microbiome

## Abstract

Several studies have compared metagenome inference performance in different human body sites; however, none specifically reported on the vaginal microbiome. Findings from other body sites cannot easily be generalized to the vaginal microbiome due to unique features of vaginal microbial ecology, and investigators seeking to use metagenome inference in vaginal microbiome research are “flying blind” with respect to potential bias these methods may introduce into analyses. We compared the performance of PICRUSt2 and Tax4Fun2 using paired 16S rRNA gene amplicon sequencing and whole-metagenome sequencing data from vaginal samples from 72 pregnant individuals enrolled in the Pregnancy, Infection, and Nutrition (PIN) cohort. Participants were selected from those with known birth outcomes and adequate 16S rRNA gene amplicon sequencing data in a case-control design. Cases experienced early preterm birth (<32 weeks of gestation), and controls experienced term birth (37 to 41 weeks of gestation). PICRUSt2 and Tax4Fun2 performed modestly overall (median Spearman correlation coefficients between observed and predicted KEGG ortholog [KO] relative abundances of 0.20 and 0.22, respectively). Both methods performed best among Lactobacillus crispatus-dominated vaginal microbiotas (median Spearman correlation coefficients of 0.24 and 0.25, respectively) and worst among Lactobacillus iners-dominated microbiotas (median Spearman correlation coefficients of 0.06 and 0.11, respectively). The same pattern was observed when evaluating correlations between univariable hypothesis test *P* values generated with observed and predicted metagenome data. Differential metagenome inference performance across vaginal microbiota community types can be considered differential measurement error, which often causes differential misclassification. As such, metagenome inference will introduce hard-to-predict bias (toward or away from the null) in vaginal microbiome research.

**IMPORTANCE** Compared to taxonomic composition, the functional potential within a bacterial community is more relevant to establishing mechanistic understandings and causal relationships between the microbiome and health outcomes. Metagenome inference attempts to bridge the gap between 16S rRNA gene amplicon sequencing and whole-metagenome sequencing by predicting a microbiome’s gene content based on its taxonomic composition and annotated genome sequences of its members. Metagenome inference methods have been evaluated primarily among gut samples, where they appear to perform fairly well. Here, we show that metagenome inference performance is markedly worse for the vaginal microbiome and that performance varies across common vaginal microbiome community types. Because these community types are associated with sexual and reproductive outcomes, differential metagenome inference performance will bias vaginal microbiome studies, obscuring relationships of interest. Results from such studies should be interpreted with substantial caution and the understanding that they may over- or underestimate associations with metagenome content.

## INTRODUCTION

Several studies have compared the performance of various metagenome inference methods in different contexts, including human body sites and environmental ecosystems (1–7). These studies consistently report that performance improves with increasing representation of taxa in reference sequence databases (16S rRNA gene and whole genome), which results in higher-quality metagenome inference for more-well-studied ecosystems (1–7). Metagenome inference methods tend to perform best for human gut and oral microbiomes and less well for other human body sites and other mammalian gut and environmental microbiomes (1–7). However, no prior studies specifically reported on metagenome inference for the vaginal microbiome (although one reported on urogenital samples but gave no additional details on these samples) (4).

Findings from other body sites cannot easily be generalized to the vaginal microbiome due to unique features of vaginal microbial ecology. For many individuals of reproductive age, the vaginal microbiome is dominated by a single *Lactobacillus* species, and these low-diversity communities are generally associated with optimal health outcomes (8–13). Conversely, diversity is very common in the gut and is considered a hallmark of gut health (14, 15). Several prevalent vaginal bacteria have only recently been characterized or remain uncharacterized (e.g., bacterial vaginosis associated bacterium 1 [BVAB1; proposed name “*Candidatus* Lachnocurva vaginae”], BVAB2, *Gardnerella* spp., Mageeibacillus indolicus, and Megasphaera lornae) (16–19). Finally, reference sequence databases contain data from >10-fold-more gut samples than vaginal samples (20). Given these differences in community composition and structure and that the vaginal microbiome is less well characterized than the gut microbiome, it is unclear how well metagenome inference methods might perform for the vaginal microbiome. It is also unclear whether these methods may introduce biases that are specific to analyses of the vaginal microbiome. To fill these gaps, we compared the performance of PICRUSt2 and Tax4Fun2 using paired 16S rRNA gene amplicon sequencing and whole-metagenome sequencing (WMGS) data from vaginal samples from pregnant individuals. We examined whether performance varied across sample characteristics (hierarchical cluster) and gene characteristics (functional category) to describe the bias PICRUSt2- and Tax4Fun2-inferred metagenomes may introduce into analyses.

## RESULTS

### Description of participants, microbiota clusters, and metagenomes.

We used data from the Pregnancy, Infection, and Nutrition (PIN) cohort to evaluate and compare the performance of PICRUSt2 and Tax4Fun2 (2, 3, 21). PIN cohort participants were recruited from prenatal clinics in North Carolina, USA, between 1995 and 2008. Participants were assigned female sex at birth and were eligible if they were ≥16 years old and at ≤29 weeks of gestation with a singleton pregnancy. Seventy-two participants were selected for this analysis from 3,063 PIN participants with live births, recorded delivery dates, and stored vaginal swabs: 35 cases who experienced early preterm birth (PTB) (<32 weeks of gestation at delivery), and 37 controls who experienced term birth (37 to 41 weeks of gestation at delivery) (see Fig. S1 in the supplemental material). Participant demographics, gestational age at sample collection, and Nugent score category at sample collection did not differ substantially between PTB cases and term birth controls (Table 1).

**TABLE 1:** PIN participant characteristics overall and by birth outcome

Characteristic	Result for participants^*a*^					
	Overall (*n* = 72)		Preterm birth cases (*n* = 35)		Term birth controls (*n* = 37)	
	*n*	%	*n*	%	*n*	%
Self-reported race						
Black	45	63	22	63	23	62
White	27	38	13	37	14	38
Age (yr)^*b*^	26	21–30	26	21–29	26	22–33
Gestational age (wk) at^*b*^:						
Sample collection	27	26–28	27	25–28	28	26–28
Delivery	37	30–40	30	28–31	40	38–41
Nugent score at sample collection^*c*^						
Non-BV (0–3)	50	69	25	71	25	68
Intermediate (4–6)	14	19	7	20	7	19
BV (7–10)	8	11	3	9	5	14
Hierarchical cluster^*d*^						
*L. crispatus* dominated	17	24	11	31	6	16
*L. iners* dominated	31	43	13	37	18	49
Mixed	24	33	11	31	13	35

a Preterm birth is <32 weeks of gestation, and term birth is between 37 and 41 weeks of gestation.

b Continuous characteristics are presented as median (*n*) and interquartile range (%).

c BV, bacterial vaginosis.

d Based on hierarchical clustering of 16S rRNA gene amplicon sequencing data.

10.1128/msystems.01003-22.2 FIG S1 STORMS checklist participant flow chart. A random subset of approximately half of the PIN participants was randomly selected to have stored vaginal swabs used for 16S rRNA gene amplicon sequencing. The observed metagenome data used in the current metagenome inference comparison were generated for a nested case-control study of the associations between the vaginal bacterial metagenome and PTB (https://doi.org/10.1128/msystems.00017-22). As race is associated with both vaginal microbiome content and preterm birth, cases and controls were frequency matched on self-reported race to address confounding. This nested metagenome case-control study was restricted to participants who reported race as Black or white due to smaller numbers of participants reporting other races and the small size of the nested case-control study (72 participants). PIN, Pregnancy, Infection and Nutrition cohort; 16S, 16S rRNA gene amplicon sequencing; PTB, preterm birth; STORMS, Strengthening the Organization and Reporting of Microbiome Studies. (Adapted from Mirzayi et al., Nat Med 27:1885–1892, 2021, https://doi.org/10.1038/s41591-021-01552-x.). Download FIG S1, PDF file, 0.1 MB. Copyright © 2023 Carter et al. 2023 Carter et al. https://creativecommons.org/licenses/by/4.0/ This content is distributed under the terms of the Creative Commons Attribution 4.0 International license.

**FIG 1:**
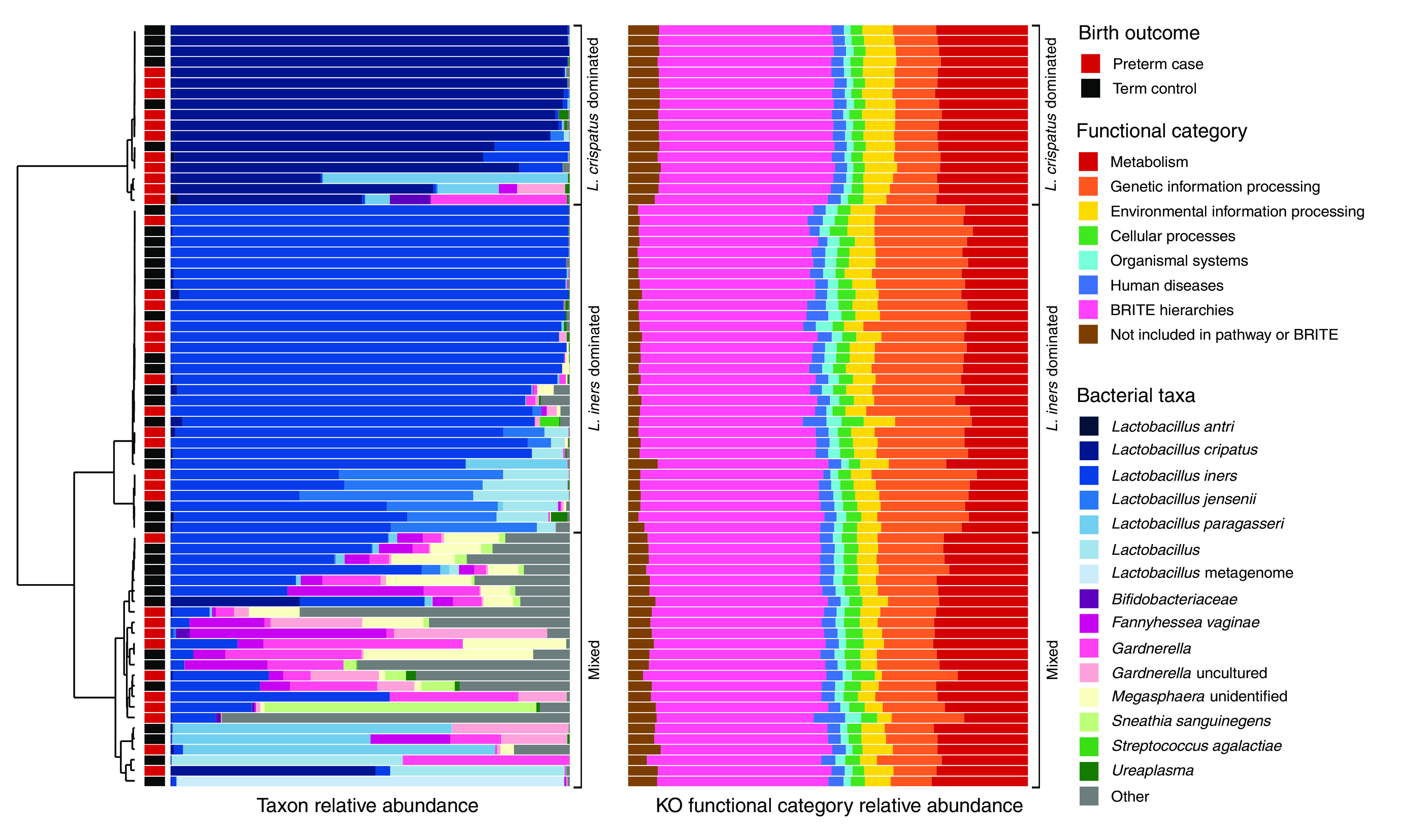
Microbiota and metagenome composition and birth outcome of 72 PIN participants included in the analysis. Shown is a dendrogram from hierarchical clustering of 16S rRNA gene amplicon sequencing data; the resulting clusters are indicated by the brackets to the right of each stacked bar plot. The taxon relative abundance from 16S rRNA gene amplicon sequencing data and the KO functional category relative abundance from observed whole-metagenome sequencing data are presented.

**FIG 2:**
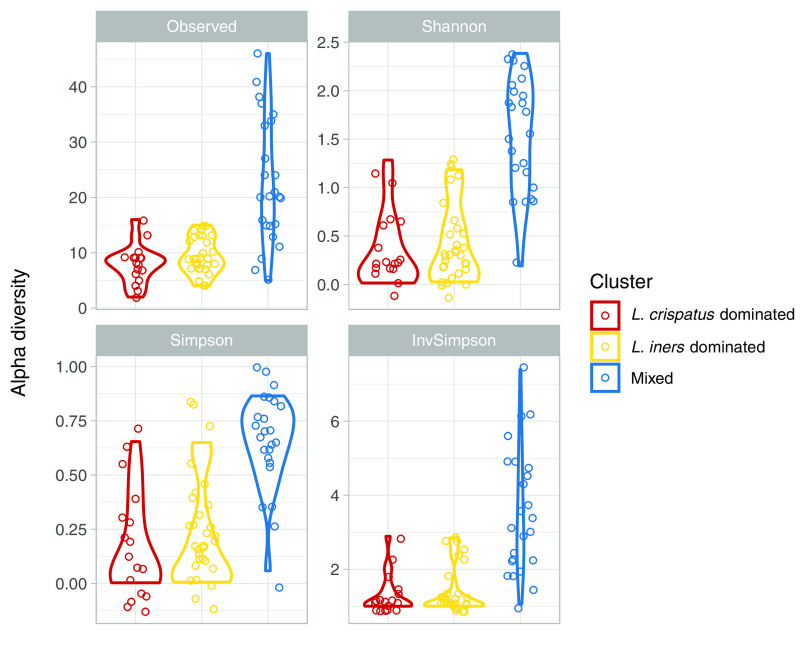
Vaginal microbiota α diversity. α diversity was estimated using 16S rRNA gene amplicon sequencing data. The α-diversity metric is indicated by the label at the top of each panel. “Observed” refers to the number of ASVs observed in a sample or the observed richness. Results were stratified and colored by hierarchical cluster from hierarchical clustering of 16S rRNA gene amplicon sequencing data.

Vaginal swabs were collected between 24 and 29 weeks of gestation, and stored swabs were used for 16S rRNA gene amplicon sequencing targeting the V1-to-V3 hypervariable regions and WMGS. The average 16S rRNA gene amplicon sequencing depth was 31,049 reads/sample (range, 804 to 54,628), and 115 amplicon sequence variants (ASVs) were assigned. In hierarchical clustering of 16S rRNA gene amplicon sequencing data using Jensen-Shannon divergence distances, we identified three clusters of vaginal microbiota composition based on *a priori* knowledge of common vaginal bacterial community types and visual inspection of the clustering dendrogram and microbiota composition stacked bar plot (Fig. 1). According to two of three internal validation statistics (connectivity and silhouette), the optimal number of clusters was two. The third internal validation statistic (Dunn) suggested the optimal number of clusters was three. With two clusters, Lactobacillus iners-dominated samples and mixed-composition samples belonged to the same cluster. Because *L. iners* dominance is commonly observed (8–12), we decided to differentiate between *L. iners*-dominated samples and mixed samples and use three clusters instead of two. Seventeen samples (24% of total) belonged to a cluster dominated by Lactobacillus crispatus, including 11 PTB cases (31% of cases). Thirty-one (43% of total) belonged to a cluster dominated by *L. iners*, including 13 PTB cases (37% of cases). Twenty-four (33% of total) belonged to a cluster of samples with mixed composition, including 11 PTB cases (31% of cases). According to all α-diversity metrics estimated, samples from the *Lactobacillus*-dominated clusters appeared to have similar distributions of α diversity (Fig. 2). The α-diversity values for samples in the mixed cluster overlapped with those from both *Lactobacillus*-dominated clusters, although the mixed cluster showed the widest ranges.

The average WMGS depth was 1,029,551 total reads/sample (range, 311,519 to 6,226,056) with 71,628 mapped reads/sample (range, 424 to 823,811) (150-bp reads), and 2,031 KEGG (Kyoto Encyclopedia of Genes and Genomes) orthologs (KOs) were assigned. At the highest level of KO functional category, observed metagenomes of samples belonging to the L. crispatus-dominated cluster appeared to be the most homogeneous, and they appeared to be more similar to metagenomes of samples belonging to the mixed cluster than to metagenomes of samples belonging to the *L. iners*-dominated cluster (Fig. 1; Fig. S2 has a stacked bar plot colored by the second-highest level of KO functional category). Compared to the other clusters, *L. iners*-dominated metagenomes appeared to be enriched with genes involved in genetic information processing and had lower relative abundances of genes involved in metabolism and uncharacterized genes. α diversity and clusters estimated from WMGS KO relative abundances are summarized and compared to estimates from 16S rRNA gene amplicon sequencing data in Text S1, Fig. S4-S7, and Table S2.

10.1128/msystems.01003-22.3 FIG S2 Metagenome composition colored by second-highest level of KO functional category and birth outcome of 72 PIN participants included in analysis. Shown is a dendrogram from hierarchical clustering of 16S rRNA gene amplicon sequencing data. The resulting clusters are indicated by the brackets to the right of the stacked bar plot. The KO functional category relative abundance from observed whole-metagenome sequencing data is presented. KO, KEGG ortholog; KEGG, Kyoto Encyclopedia of Genes and Genomes. Download FIG S2, PDF file, 0.04 MB. Copyright © 2023 Carter et al. 2023 Carter et al. https://creativecommons.org/licenses/by/4.0/ This content is distributed under the terms of the Creative Commons Attribution 4.0 International license.

**FIG 3:**
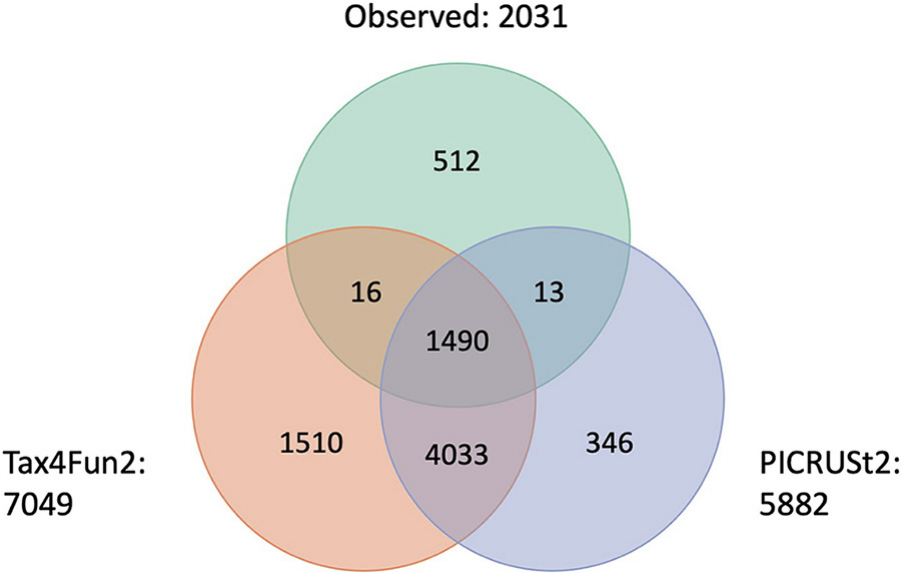
Number and overlap of KOs observed and predicted. “Observed” refers to KOs annotated by HUMAnN2 in the whole-metagenome sequencing data.

We inferred metagenome content from 16S rRNA gene amplicon sequencing data using PICRUSt2 and Tax4Fun2 according to the developers’ recommendations (2, 3). PICRUSt2 predicted the presence of 5,882 KOs, of which 1,503 (26%) were observed in WMGS data (Fig. 3). Tax4Fun2 predicted the presence of 7,049 KOs, of which 1,506 (21%) were observed. A total of 1,490 KOs were observed in WMGS data and predicted by PICRUSt2 and Tax4Fun2, and all evaluations and comparisons of metagenome inference performance were restricted to these 1,490 KOs.

### Metagenome inference performance varied by dominant *Lactobacillus* species.

We evaluated metagenome inference performance using two approaches. First, we estimated KO-specific Spearman correlation coefficients between observed and predicted KO relative abundances. Second, we estimated Spearman correlation coefficients between univariable hypothesis test *P* values estimated using observed and predicted KO relative abundances. This approach was proposed by Sun et al. following their observation that Spearman correlation coefficients between observed and predicted KO relative abundances are insensitive to randomly permutating the data, indicating they may be an unreliable measure of metagenome inference performance (6). We used Wilcoxon tests to test the null hypothesis that KO relative abundances do not differ between PTB cases and term birth controls. We performed Wilcoxon tests using observed and predicted KO relative abundances separately, and we transformed *P* values according to the following equation to capture the significance and direction of KO relative abundance differences:

$$P_{t}={\text{log}}_{\text{10}}\left(P\right)\times \text{sign [}\left(\bar{\text{KO}}\text{|PTB}\right)-\left(\bar{\text{KO}}\text{|term birth}\right)]$$

In this equation, *P_t_* is the transformed *P* value, *P* is the Wilcoxon test *P* value, $\bar{\text{KO}}\text{|PTB}$ is the mean KO relative abundance among PTB cases, and $\bar{\text{KO}}\text{|term birth}$ is the mean KO relative abundance among term birth controls. We performed all analyses with the original observed and predicted metagenome data, as well as with 100 random permutations of the observed and predicted metagenome data in which KO relative abundances were independently permuted across samples, which serves as a robustness check (6).

**FIG 4:**
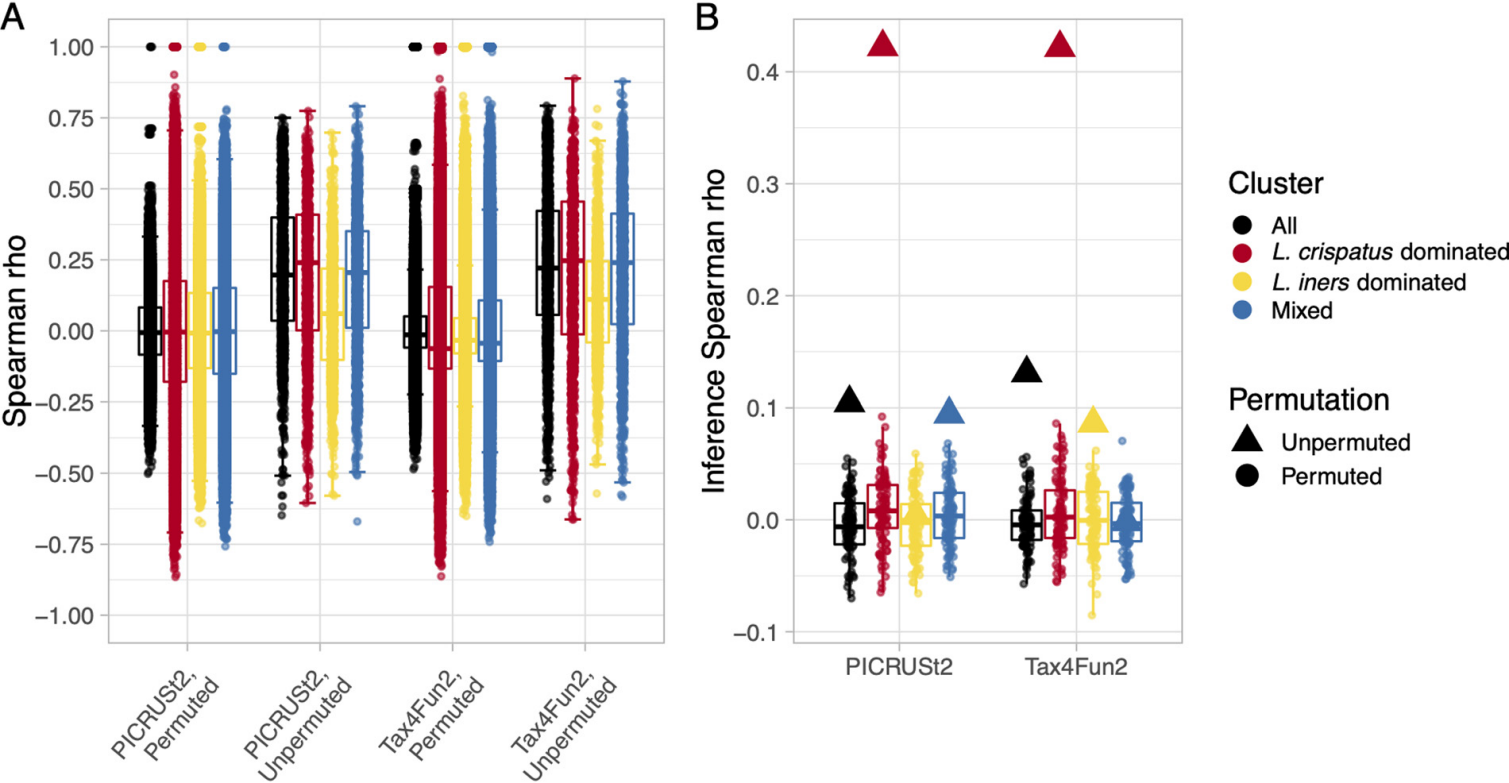
Vaginal microbiome metagenome inference performance varies across hierarchical cluster. Shown are clusters from hierarchical clustering of 16S rRNA gene amplicon sequencing data. Analyses were performed with the original observed and predicted metagenome data and with 100 random permutations of observed and predicted metagenome data (KO relative abundances independently permuted across samples), which serves as a robustness check. Spearman correlation coefficients between observed and predicted KO relative abundances, stratified by hierarchical cluster, metagenome inference method, and permutation, are shown in panel A. Spearman correlation coefficients between univariable hypothesis test transformed *P* values estimated with observed and predicted KO relative abundances, stratified by hierarchical cluster, metagenome inference method, and permutation, are shown in panel B. We used Wilcoxon tests to test the null hypothesis that KO relative abundances do not differ between PTB cases and term birth controls. We performed Wilcoxon tests using observed and predicted KO relative abundances separately, and we transformed *P* values according to the equation from the section “Metagenome inference performance varied by dominant *Lactobacillus* species” to capture the significance and direction of KO relative abundance differences.

The median Spearman correlation between observed and PICRUSt2-predicted KO relative abundances was 0.20 (range, −0.65 to 0.75) (Fig. 4A). We stratified by hierarchical cluster to evaluate whether PICRUSt2 or Tax4Fun2 performance varied across common vaginal microbiota community types. The median correlation was similar among the L. crispatus-dominated (0.24) and mixed (0.21) clusters and lower among the *L. iners*-dominated cluster (0.06). We observed a similar pattern for the Tax4Fun2 predictions, although median correlations were higher (overall, 0.22 [range, −0.59 to 0.79]; L. crispatus-dominated, 0.25; mixed, 0.24; *L. iners*-dominated, 0.11). Correlations were not robust to permutation (median correlations of ~0 for permuted data), indicating this may be a reliable method for evaluating metagenome inference performance for the vaginal microbiome.

For the hypothesis test comparing KO relative abundances between cases and controls, transformed *P* value correlations were highest among L. crispatus-dominated samples (PICRUSt2, 0.42; Tax4Fun2, 0.42) (Fig. 4B). Correlations were low overall (PICRUSt2, 0.10; Tax4Fun2, 0.13) among *L. iners*-dominated samples (PICRUSt2, 0.00; Tax4Fun2, 0.09) and among mixed samples (PICRUSt2, 0.09; Tax4Fun2, 0.00). Correlations were not robust to permutation, and variation across clusters did not appear to be influenced by transformed *P* value outliers (Fig. S3). Taken together, these data indicate that PICRUSt2 and Tax4Fun2 perform best among L. crispatus-dominated microbiotas and poorly among *L. iners*-dominated microbiotas.

10.1128/msystems.01003-22.4FIG S3Scatterplots of transformed *P* values estimated from observed and predicted KO relative abundances. Plots are colored by cluster from hierarchical clustering of 16S rRNA gene amplicon sequencing data in panel A or colored by KO functional category in panel B. We used Wilcoxon tests to test the null hypothesis that KO relative abundances do not differ between PTB cases and term birth controls. We performed Wilcoxon tests using observed and predicted KO relative abundances separately, and we transformed *P* values according to the following equation in order to capture the significance and direction of KO relative abundance differences:
$$P_{t}={\text{log}}_{\text{10}}\left(P\right)\times \text{sign [}\left(\bar{\text{KO}}\text{|PTB}\right)-\left(\bar{\text{KO}}\text{|term birth}\right)]$$
In the equation, *P_t_* is the transformed *P* value, *P* is the Wilcoxon test *P* value, $\bar{\text{KO}}\text{|PTB}$ is the mean KO relative abundance among PTB cases, and $\bar{\text{KO}}\text{|term birth}$ is the mean KO relative abundance among term birth controls. KO, KEGG ortholog; PTB, preterm birth; KEGG, Kyoto Encyclopedia of Genes and Genomes. Download FIG S3, PDF file, 0.9 MB.Copyright © 2023 Carter et al.2023Carter et al.https://creativecommons.org/licenses/by/4.0/This content is distributed under the terms of the Creative Commons Attribution 4.0 International license.

**FIG 5:**
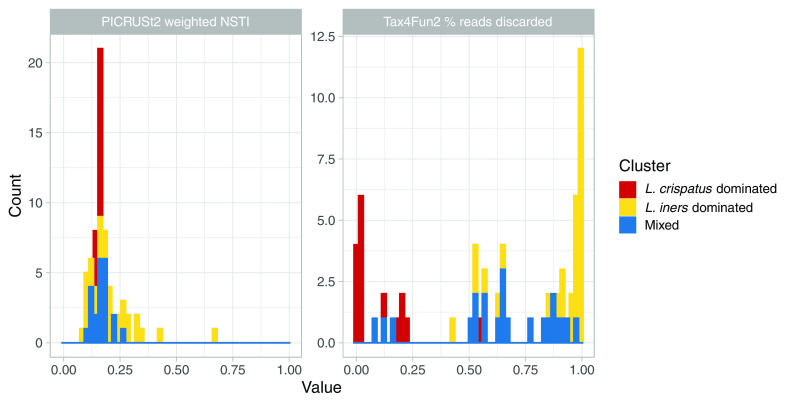
ASVs observed in *L. iners*-dominated samples are less well represented in metagenome inference reference databases. Shown are clusters from hierarchical clustering of 16S rRNA gene amplicon sequencing data. The weighted NSTI (nearest-sequenced-taxon index) was estimated by PICRUSt2 as a sample-specific measure of relatedness between observed ASVs and their nearest neighbors in the PICRUSt2 reference database, weighted by sample composition. Lower values indicate an ASV is more closely related to its nearest neighbor. In Tax4Fun2, ASVs that are unrepresented in the reference database and that have <97% ANI to their nearest reference neighbor’s 16S rRNA gene are discarded prior to metagenome inference.

PICRUSt2 provides a weighted nearest-sequenced-taxon index (wNSTI), a sample-specific measure of relatedness between observed ASVs and their nearest neighbors in the PICRUSt2 reference database, weighted by sample composition (3). The median wNSTI overall was 0.16, and while medians were similar among L. crispatus- and *L. iners*-dominated samples (0.16 and 0.20, respectively), *L. iners*-dominated samples showed much wider range of values and substantial density of samples at values greater than the maximum wNSTI for the L. crispatus cluster (0.17) (Fig. 5). In Tax4Fun2, ASVs that are unrepresented in the reference database and that have <97% average nucleotide identity (ANI) to their nearest neighbor are discarded prior to metagenome inference (2). The median proportion of discarded reads was 66% overall, with 3% discarded among L. crispatus-dominated samples and 97% discarded among *L. iners*-dominated samples (Fig. 5).

### Metagenome inference performance varied across KO function.

**FIG 6:**
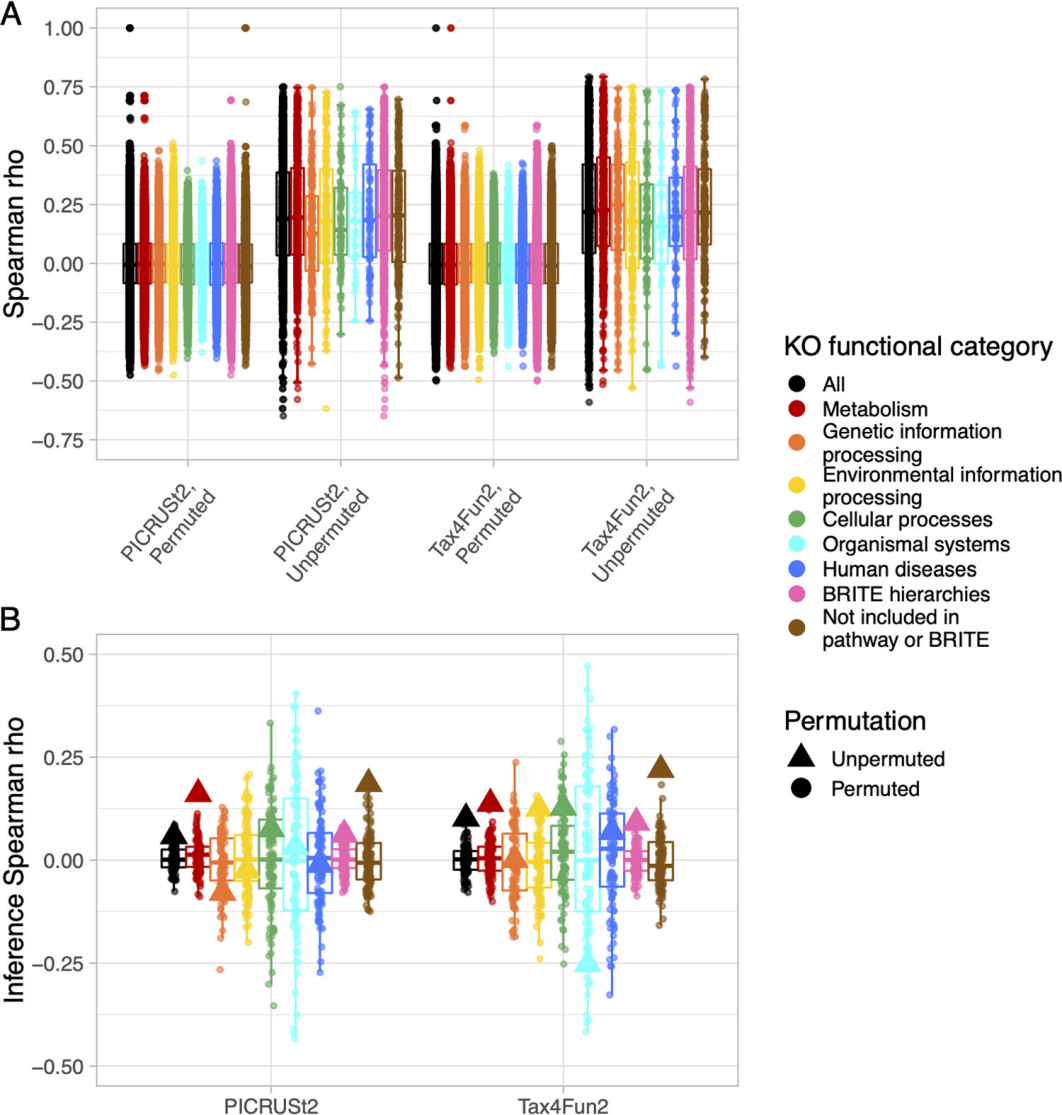
Vaginal microbiome metagenome inference performance varies across KO functional category. Correlation analyses were performed with original observed and predicted metagenome data and with 100 random permutations of observed and predicted metagenome data (KO relative abundances independently permuted across samples), which serves as a robustness check. Spearman correlation coefficients between observed and predicted KO relative abundances, stratified by KO functional category, metagenome inference method, and permutation, are shown in panel A. Spearman correlation coefficients between univariable hypothesis test transformed *P* values estimated with observed and predicted KO relative abundances, stratified by KO functional category, metagenome inference method, and permutation, are shown in panel B. We used Wilcoxon tests to test the null hypothesis that KO relative abundances do not differ between PTB cases and term birth controls. We performed Wilcoxon tests using observed and predicted KO relative abundances separately, and we transformed *P* values according to the equation from the section “Metagenome inference performance varied by dominant *Lactobacillus* species” in order to capture the significance and direction of KO relative abundance differences.

We also stratified by the highest level of KO functional category to evaluate whether PICRUSt2 or Tax4Fun2 performance varied across groups of related genes. Median Spearman correlations between observed and PICRUSt2-predicted KO relative abundances were similar across KO functional categories (range from 0.13 for genetic information processing to 0.20 for BRITE hierarchies and uncharacterized) (Fig. 6A). We also observed little variation in correlations for Tax4Fun2 predictions, though correlations were slightly higher (range from 0.16 for organismal systems to 0.25 for genetic information processing). Correlations were not robust to permutation.

We observed more variation across KO functional categories in correlations between transformed *P* values estimated with observed and predicted KO relative abundances (Fig. 6B). For the hypothesis test comparing KO relative abundances between cases and controls, correlations were highest for uncharacterized KOs and metabolism (PICRUSt2, 0.18 and 0.16, respectively; Tax4Fun2, 0.22 and 0.14, respectively). PICRUSt2 performed worst for genetic information processing (−0.08), and Tax4Fun2 performed worst for organismal systems (−0.25). Correlations were not robust to permutation, and variation across KO functional categories did not appear to be influenced by transformed *P* value outliers (Fig. S3). These data indicate that PICRUSt2 and Tax4Fun2 may perform best for uncharacterized genes and poorly for genes involved in genetic information processing.

### *L. crispatus*-dominated metagenomes were enriched with good-performance KOs, and *L. iners*-dominated metagenomes were enriched with poor-performance KOs.

**FIG 7:**
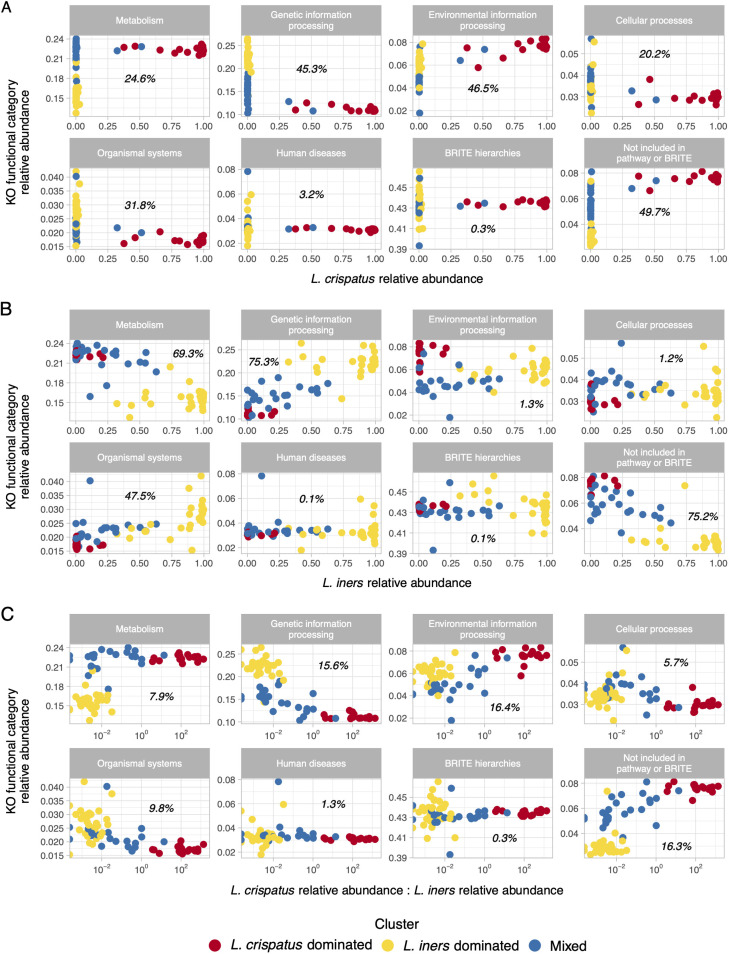
Correlations between KO functional category relative abundances and *L. iners* and L. crispatus relative abundances. Scatterplots of L. crispatus relative abundance versus KO functional category relative abundance with one panel for each KO functional category are shown in panel A, *L. iners* relative abundance versus KO functional category relative abundance with one panel for each KO functional category is shown in panel B, and the ratio of L. crispatus relative abundance to *L. iners* relative abundance versus KO functional category relative abundance with one panel for each KO functional category is shown in panel C. The italicized text in each panel presents the *R*^2^ value from a linear model regressing KO functional category relative abundance (response variable) on L. crispatus relative abundance (A), *L. iners* relative abundance (B), or the ratio of L. crispatus relative abundance to *L. iners* relative abundance (C) (explanatory variables). The *R*^2^ values represent the proportion of variation in KO relative abundance explained by the variation in L. crispatus relative abundance (A), *L. iners* relative abundance (B), or the ratio of L. crispatus relative abundance to *L. iners* relative abundance (C). Points are colored by hierarchical cluster from hierarchical clustering of 16S rRNA gene amplicon sequencing data.

We examined differences in observed metagenome content between L. crispatus- and *L. iners*-dominated samples as a potential cause of differential metagenome inference performance between these clusters. L. crispatus relative abundance was strongly positively correlated with uncharacterized KO relative abundance (linear model, *R*^2^ = 50%), whereas *L. iners* relative abundance was strongly negatively correlated with uncharacterized KO relative abundance (linear model, *R*^2^ = 75%) (Fig. 7). L. crispatus relative abundance was strongly negatively correlated with genetic information processing KO relative abundance (linear model, *R*^2^ = 45%), while *L. iners* relative abundance was strongly positively correlated with genetic information processing KO relative abundance (linear model, *R*^2^ = 75%) (Fig. 7).

## DISCUSSION

While the performance of various metagenome inference methods have been compared in several human body site and environmental ecosystems, to our knowledge this is the first study specific to the vaginal microbiome (1–7). Our cluster-stratified analysis demonstrated that PICRUSt2 and Tax4Fun2 perform best among L. crispatus-dominated communities and poorly among *L. iners*-dominated communities. We attribute this differential performance to biological differences between the communities, as well as reference differences between the communities. In terms of biological difference, our KO functional category-stratified analysis highlighted that *L. iners*-dominated microbiotas tend to be enriched with KO functional categories that are inferred poorly, namely, genetic information processing. This is consistent with a prior genomic comparison of 15 *L. iners* and 15 L. crispatus strains. The *L. iners* core and accessory genomes contained larger proportions of genes involved in replication and repair, transcription, and translation than the L. crispatus core and accessory genomes (22). Within their respective clusters in the current analysis, *L. iners* and L. crispatus dominated communities with averages of 86% and 87% relative abundance, respectively. It follows that differences in genome composition between the species will yield differences in metagenome composition between *L. iners*- and L. crispatus-dominated communities (in general and with respect to genetic information processing genes). This appears to be a driver of poor metagenome inference performance among *L. iners*-dominated communities. It is unclear why PICRUSt2 and Tax4Fun2 performed poorly for genetic information processing genes. One prior function-stratified analysis of gut microbiome samples found that the original implementation of PICRUSt, PICRUSt2, and the original implementation of Tax4Fun tended to perform well for genetic information processing genes, with the exception of genes involved in transcription (6).

A second biological difference is related to interstrain variation. Marker gene sequencing is largely incapable of resolving taxa to the strain/subspecies level. Instead, heterogeneous strains are collapsed into a single higher-order feature, and interstrain variation is structurally removed from the data. If we cannot identify strains in 16S rRNA gene amplicon sequencing data, we cannot infer strain-specific genome content, and interstrain differences in gene content are structurally removed from inferred metagenome data. *L. iners* is estimated to have more interstrain variation than other vaginal lactobacilli (22–25), so this limitation will more seriously impact gene content inference for *L. iners* and metagenome inference for *L. iners*-dominated communities than it will for L. crispatus.

In terms of reference differences between *L. iners*- and L. crispatus-dominated communities, *L. iners* is less well represented than L. crispatus in the reference databases used in the current analysis. HUMAnN2 was used to characterize gene families in the observed WMGS data, and the HUMAnN2 protein reference database was generated from UniRef90 and UniRef50 (prior to 2017, it was unclear which release) (26). As of 31 December 2016, UniRef90 contained 13,078 L. crispatus proteins and 3,205 *L. iners* proteins, and UniRef50 contained 6,146 L. crispatus proteins and 1,677 *L. iners* proteins. L. crispatus genomes are ~1.5- to 2-fold larger and contain ~1.5- to 2-fold more predicted coding sequences than *L. iners* genomes (22, 24); however, there are 4.1-fold more and 3.7-fold more L. crispatus proteins in UniRef90 and UniRef50, respectively. *L. iners* is substantially underrepresented in UniRef90/UniRef50, so it is similarly underrepresented in the HUMAnN2 protein reference database. KO sequences uniquely originating from *L. iners* are less likely to be identified in our data than those uniquely originating from L. crispatus. However, this bias does not directly impact our correlation analysis results. Because this bias results in underdetection of *L. iners* KOs in the observed data, there are no “gold standard” data for these KOs, and no comparison can be made with inferred metagenome data if the KOs are predicted to be present.

The PICRUSt2 reference database was constructed using bacterial genomes in the Integrated Microbial Genomes & Microbiomes database on 8 November 2017 (3), which contained 15 L. crispatus genomes and 17 *L. iners* genomes. Despite similar representation, our observed L. crispatus ASV was more closely related to its nearest reference neighbor than our observed *L. iners* ASV was to its nearest reference neighbor (Fig. 4). (Neither ASV was distantly related enough to be discarded prior to metagenome inference). Unlike for HUMAnN2, bias introduced by the PICRUSt2 reference database differentially impacts L. crispatus- and *L. iners*-dominated communities, contributing to poorer performance for *L. iners*-dominated communities. The Tax4Fun2 reference database was constructed using all complete genomes in NCBI RefSeq on 18 August 2018 (2), which contained 6 L. crispatus genomes and 0 *L. iners* genomes. Tax4Fun2 identifies each observed ASV’s nearest neighbor in its reference database and infers gene content for ASVs that have ≥97% sequence identity to their nearest neighbor; the remaining ASVs and associated reads are discarded (2). Without reference data for *L. iners*, >41% of reads were discarded from each *L. iners*-dominated sample prior to metagenome inference, and 75% of *L. iners*-dominated samples had ≥90% of reads discarded (Fig. 4). Among L. crispatus-dominated samples, most had <25% of reads discarded, and at most 55% of reads were discarded. Clearly, the *L. iners* ASV we observed is not sufficiently closely related to its nearest reference neighbor for it to contribute to Tax4Fun2 metagenome inference. Despite massive loss of reads from *L. in*ers-dominated samples in Tax4Fun2 and no loss in PICRUSt2, Tax4Fun2 performed somewhat better for *L. iners*-dominated communities, which likely reflects differences in accuracy of genome content inference for other taxa present.

Taking an epidemiologic lens to these results, we can consider differential metagenome inference performance to be analogous to differential measurement error, resulting in differential misclassification. Differential measurement error occurs when the degree or direction of outcome (response variable) measurement error differs across exposure (explanatory variable) statuses, or when the degree or direction of exposure measurement error differs across outcome statuses. Differential measurement error and subsequent differential misclassification bias effect estimates, and this bias is hard to predict in that it can be in either direction (toward or away from the null value) (27, 28). On the other hand, nondifferential measurement error and nondifferential misclassification (independent of exposure or outcome status) typically bias effect estimates toward the null (27, 28). To qualify as differential measurement error, metagenome inference performance must differ across exposure or outcome status or across a factor that is associated with exposure or outcome status. While cluster or community type will not be the exposure or outcome of interest in every vaginal microbiome study that uses metagenome inference, we posit that cluster will be associated with the exposure or the outcome in virtually all of these studies based on the large body of evidence supporting such associations (9, 10, 12, 29–31). It follows that metagenome inference will introduce differential measurement error, differential misclassification, and hard-to-predict bias in vaginal microbiome research. Results from these analyses should be interpreted with substantial caution and the understanding that they may over- or underestimate associations with metagenome content. This is particularly concerning given the high global prevalence of *L. iners*-dominated vaginal microbiotas (8–12); poor metagenome inference among these communities will impact similarly large proportions of study populations.

Several studies have compared the performance of various metagenome inference methods using gut microbiome samples, and we can use these reports to benchmark PICRUSt2 and Tax4Fun2 performance for the vaginal microbiome. We will consider results based on observed and predicted KO relative abundance correlations because few studies have examined *P* value correlations (5, 6), and assessing performance based on *P* value correlations depends on the hypothesis tested and the true effect size. Among stool samples from various populations, four analyses of six metagenome inference methods report median correlations of ~0.60 to 0.90 (3–6). In our analysis, median relative abundance correlations were 0.19 and 0.22 (Fig. 4A and Fig. 5A), indicating substantially worse performance for the vaginal microbiome than for the gut microbiome. Poorer metagenome inference for the vaginal microbiome can be attributed, likely in large part, to the vaginal microbiome being relatively understudied compared to the gut microbiome. A recent investigation of all human microbiome data sets in the Sequence Read Archive, DNA Data Bank of Japan, and European Nucleotide Archive revealed that 220,017 (49%) of 444,829 samples were from the gut, compared to 17,784 (4%) from the vagina (20). Given its prevalence (8–12), underrepresentation of *L. iners* in metagenome inference reference databases contributes to poorer performance for the vaginal microbiome than the gut. Additionally, a taxon formerly considered one species, Gardnerella vaginalis, was recently identified to the species level as four named species (Gardnerella leopoldii, Gardnerella piotii, Gardnerella swidsinskii, and *G. vaginalis*) and 9 genomic species (19). *Gardnerella* species cannot be differentiated by 16S rRNA gene sequences, so the genomic diversity of *Gardnerella* species cannot be captured by metagenome inference. Considering the relative abundance of *Gardnerella* spp. was as high as 50% in this analysis (an average of 18% within the mixed cluster), inability to accurately capture *Gardnerella* species’ genome content will decrease metagenome inference performance.

Our results also indicate poorer metagenome inference performance for the vaginal microbiome than has been reported for the urogenital microbiome. One study examined the first implementations of PICRUSt and Tax4Fun using urogenital samples and reported median relative abundance correlation of ~0.65 (4). Reasons for this discrepancy are unclear because additional details on the urogenital samples were not reported.

Our analysis has several strengths. This is the first published metagenome inference comparison specific to the vaginal microbiome. Focusing on the vaginal microbiome enabled us to identify patterns of metagenome inference performance that directly relate to vaginal microbial ecology, as well as important implications for risk of bias in vaginal microbiome research that employs metagenome inference. We were able to confidently identify this pattern because we consistently observed differential metagenome inference performance across clusters in both of the evaluations we performed (relative abundance and *P* value correlations). Using both evaluation methods is a second strength of this analysis. Finally, 16S rRNA gene amplicon sequencing and WMGS depths achieved in the parent study indicate the high quality of the sequencing data used.

Our findings should also be interpreted in the context of the study’s limitations. With 72 participants, we identified only three clusters of vaginal microbiota composition. With a larger population, we may have been able to characterize metagenome inference performance for more vaginal microbiota community types observed in U.S. populations (e.g., Lactobacillus gasseri dominated, Lactobacillus jensenii dominated, subgroups of *Lactobacillus* dominance, and diverse bacterial vaginosis [BV]-like communities) (30). Second, all participants were pregnant and enrolled in North Carolina, USA. This limits generalizability of our results to nonpregnant populations and populations in other regions, especially considering documented differences in vaginal microbiota composition by pregnancy status (32–35) and between global populations (36–40). Finally, this analysis was restricted to metagenome features that were observed and predicted. Totals of 541 (27%) observed KOs, 4,392 (75%) PICRUSt2-predicted KOs, and 5,559 (79%) Tax4Fun2-predicted KOs did not contribute to the analysis (Fig. 3).

Our analysis demonstrates that metagenome inference methods perform more poorly for the vaginal microbiome than for the gut microbiome. Performance was differential according to vaginal microbiota cluster, with the best performance among L. crispatus-dominated communities and the worst among *L. iners*-dominated communities. Genome content differences between *L. iners* and L. crispatus and underrepresentation of *L. iners* in reference databases appear to drive differential performance, which will result in differential measurement error, differential misclassification, and hard-to-predict bias in vaginal microbiome research that employs metagenome inference. As the cost of whole-metagenome sequencing continues to fall, investigators will ideally be able to characterize the actual vaginal bacterial metagenome, obviating the need for and eliminating measurement error introduced by metagenome inference.

## MATERIALS AND METHODS

We used paired 16S rRNA gene amplicon sequencing and WMGS data from the PIN cohort to evaluate and compare the performance of PICRUSt2 and Tax4Fun2 for the vaginal microbiome (2, 3, 21). The PIN cohort is a prospective cohort study approved by the University of North Carolina Institutional Review Board (protocol 16-2166, with continuous approval since August 2016), and all participants provided written informed consent prior to enrollment. For this secondary analysis, we selected participants from among those who consented to additional testing of stored specimens. We report our results according to the Strengthening The Organization and Reporting of Microbiome Studies (STORMS) guidelines (Table S1) (41).

### PIN participants and study procedures.

PIN cohort participants were recruited from prenatal clinics at the Wake County Human Services Department, WakeMed Medical Center/Wake Area Health Education Center, and University of North Carolina hospitals in North Carolina between 1995 and 2008. Potential participants were eligible if they were assigned female sex at birth, ≥16 years old, and at ≤29 weeks of gestation with a singleton pregnancy. Potential participants were ineligible if they did not plan to continue care or deliver at the study site, did not have telephone access, or were non-English speaking. Study staff identified potential participants through medical record review and approached potential participants about enrolling.

Participants were enrolled at ≤29 weeks of gestation and followed until delivery. Demographic characteristics were recorded at enrollment, and clinicians collected two Dacron-tipped swabs from the posterior vaginal apex between 24 and 29 weeks of gestation. One swab was used for evaluation of BV according to Nugent score and then placed in a Digene Virapap tube with transport medium (Digene Diagnostics, Inc., Silver Spring, MD) (42). The second swab was placed in a Roche Amplicor specimen collection tube containing extraction buffer (Roche Diagnostic Systems, Inc., Branchburg, NJ). Specimens were refrigerated prior to transport and frozen at −70°C within 6 h of collection. Following enrollment, a telephone questionnaire was used to collect information on reproductive and medical history—sexual behaviors during pregnancy and tobacco, alcohol, and other drug use. Gestational age at delivery was defined by early ultrasound (completed <20 weeks of gestation) for 90% of participants and by last menstrual period date for the remaining participants. Birth outcomes were abstracted from medical records.

Participants were selected for this substudy in a nested case-control design. Cases were participants who experienced early preterm birth at <32 weeks of gestation, and controls were randomly selected from among participants who experienced term birth between 37 and 41 weeks of gestation. Cases and controls were selected from among participants with adequate extracted DNA for WMGS and ≥800 high-quality reads from 16S rRNA gene amplicon sequencing.

### Microbiome data generation.

Stored vaginal swabs were used for 16S rRNA gene amplicon sequencing as previously reported (43–45). Frozen swabs were thawed on ice, DNA was extracted using the PowerSoil DNA isolation kit (Qiagen, Hilden, Germany) according to manufacturer recommendations, and extracted DNA was quantified using PicoGreen. Extracted DNA was amplified with barcoded primers targeting the 16S rRNA gene V1-to-V3 hypervariable regions using protocols established by the Virginia Commonwealth University Vaginal Human Microbiome Project (46). The primers contain an Illumina linkage adaptor, unique barcode (8 bases for forward primer, 12 for reverse), a variable sequence spacer (0 to 6 bases), and 16S rRNA gene primers (full primer sequences are available in reference 47). The forward primer was a 4:1 mixture of primers Fwd-P1 and Fwd-P2. The reverse primer was Rev1B (46, 47). Amplicon samples were multiplexed (384 samples/run) using a sample-specific dual-index strategy and sequenced on the Illumina MiSeq platform (2 × 300 base-paired-end protocol) (Illumina, San Diego, CA). Resulting amplicon sequence data were processed using QIIME2 (version 2019.1.0) and the DADA2 denoise-single method, truncating reads at 120 bases (48, 49). Taxonomy was assigned using the Ribosomal Database Project Naïve Bayesian Classifier and SILVA database (release 138.1) (50–52).

Extracted DNA was also used for whole-metagenome library preparation. Paired-end metagenomic DNA libraries were prepared from 250 ng of genomic DNA using the Accel-NGS 2S Plus DNA library kit (Integrated DNA Technologies, Coralville, IA) with an insert size of ~350 bp. Samples were preprocessed as previously described (44, 45). Barcoded libraries were multiplexed (10 to 11 samples/lane) and sequenced on the Illumina HiSeq 4000 (2 × 150-base-paired-end reads). Raw sequences were demultiplexed using Illumina’s bcl2fastq software and quality filtered using MEEPTOOLS (53, 54), where reads shorter than 70 bases and with a MEEP quality score of >1 were excluded. WMGS data were screened for duplicate reads, and human sequences were removed by aligning reads to the hg19 build of the human genome using the BWA aligner (55). The remaining reads were processed and aligned according to the approach recommended by Martin and colleagues (56). Reads with <60 nonmasked, non-low-complexity bases were considered low complexity and discarded (56). The remaining reads were aligned using the CLC Assembly Cell aligner (Qiagen, Hilden, Germany) requiring 80% identity over 75% of the query length (56). Gene families and functional pathways were characterized using HUMAnN2 and the KEGG Orthology database (26, 57–59).

### Statistical analysis.

We used descriptive statistics to summarize participant characteristics, including maternal age, self-reported race, and gestational ages at vaginal swab collection and delivery. We estimated vaginal microbiota α diversity based on 16S rRNA gene amplicon sequencing data using the estimate_richness function of the phyloseq package (v.1.34.0 throughout) in R (v.4.0.4 throughout). We categorized vaginal microbiota composition by hierarchical clustering of 16S rRNA gene amplicon sequencing data based on Jensen-Shannon divergence distances and Ward linkage using the distance function of the phyloseq package, hclust and cutree functions of the stats package (v.4.0.4), and dendsort function of the dendsort package (v.0.3.4) in R. We used the same functions to estimate α diversity and identify clusters based on WMGS KO relative abundances, and we compared α diversity and cluster membership between 16S rRNA gene amplicon sequencing and WMGS data.

We inferred metagenome content from 16S rRNA gene amplicon sequencing data using PICRUSt2 and Tax4Fun2 according to the developers’ recommendations/default parameters (2, 3). We selected PICRUSt2 and Tax4Fun2 because their original implementations, PICRUSt and Tax4Fun, are highly cited among metagenome inference methods (as of 15 February 2023, PICRUSt and Tax4Fun original publications were cited 7,309 and 1,119 times, respectively) (1, 4). We selected their second implementations, PICRUSt2 and Tax4Fun2, because they demonstrated marked performance improvements over the original implementations (1–4). We implemented PICRUSt2 (v.2.4.1) in Miniconda (v.4.9.2). In addition to inferred metagenome composition, PICRUSt2 computes wNSTI, a sample-specific measure of relatedness between observed ASVs and their nearest neighbors in the PICRUSt2 reference database, weighted by sample composition (3). We implemented Tax4Fun2 with the default reference database and 99% clustering threshold using the runRefBlast and makeFunctionalPrediction functions of the Tax4Fun2 package (v.1.1.5) in R. For ASVs unrepresented in the Tax4Fun2 reference database, the nearest neighbor in the database is identified, and the ASVs are assumed to have the same gene content as their nearest neighbor when the 16S rRNA gene ANI is ≥97% (2). ASVs with a <97% ANI are discarded prior to metagenome inference (2). Tax4Fun2 provides the proportion of ASVs and reads discarded.

For metagenome inference performance evaluation and comparison, we restricted the observed, PICRUSt2-inferred, and Tax4Fun2-inferred metagenome data sets to KOs present at relative abundance of >0% in all three data sets. We evaluated metagenome inference performance using two approaches. First, we estimated KO-specific Spearman correlation coefficients between observed and predicted KO relative abundances. Second, we estimated Spearman correlation coefficients between univariable hypothesis test *P* values estimated using observed and predicted KO relative abundances. This approach was proposed by Sun et al. following their observation that Spearman correlation coefficients between observed and predicted KO relative abundances are insensitive to randomly permutating the data, indicating they may be an unreliable measure of metagenome inference performance (6). We used Wilcoxon tests to test the null hypothesis that KO relative abundances do not differ between PTB cases and term birth controls. We performed Wilcoxon tests using observed and predicted KO relative abundances separately, and we transformed *P* values according to the equation shown above in the section “Metagenome inference performance varied by dominant *Lactobacillus* species” to capture the significance and direction of KO relative abundance differences.

For both evaluation approaches, we performed two stratified analyses to evaluate whether metagenome inference performance differs according to vaginal microbiota hierarchical cluster (from 16S rRNA gene amplicon sequencing) or KO functional category (highest-level categories). We performed each analysis with the original observed and predicted metagenome data, as well as with 100 random permutations of the observed and predicted metagenome data in which KO relative abundances were independently permuted across samples, which serves as a robustness check (6). We used linear model *R*^2^ values to examine relationships between KO functional category relative abundances (response variables) and L. crispatus relative abundances, *L. iners* relative abundances, and the ratio of L. crispatus to *L. iners* relative abundances (explanatory variables). We examined PICRUSt2 wNSTI values and the proportion of reads discarded in Tax4Fun2 as additional measures of sample-specific performance.

### Data availability.

The 16S and shotgun metagenome sequencing data can be accessed at the NCBI Sequence Read Archive (SRA) under BioProject no. PRJNA876771 (https://www.ncbi.nlm.nih.gov/bioproject/PRJNA876771/). The R script, R data files, and corresponding codebook/data dictionary used in the analysis are available on GitHub (https://github.com/kaycart/mg-inference-comp).

10.1128/msystems.01003-22.1 TEXT S1 Results from hierarchical clustering of whole-metagenome sequencing KO relative abundance data and α-diversity estimates for whole-metagenome sequencing KO relative abundance data. Also compared are the cluster membership and α diversity estimated from 16S rRNA gene amplicon sequencing data and whole-metagenome sequencing data. Download Text S1, PDF file, 0.01 MB. Copyright © 2023 Carter et al. 2023 Carter et al. https://creativecommons.org/licenses/by/4.0/ This content is distributed under the terms of the Creative Commons Attribution 4.0 International license.

10.1128/msystems.01003-22.6 FIG S4 Microbiota and metagenome composition and birth outcome of 72 PIN participants included in the analysis, ordered by whole-metagenome sequencing hierarchical clustering dendrogram. Shown is a dendrogram from hierarchical clustering of WMGS KO relative abundance data. The resulting WMGS clusters are indicated by the brackets to the right of each stacked bar plot. The taxon relative abundance from 16S rRNA gene amplicon sequencing data and KO functional category relative abundance from observed whole-metagenome sequencing data are presented. WMGS, whole-metagenome sequencing; KO, KEGG ortholog; KEGG, Kyoto Encyclopedia of Genes and Genomes. Download FIG S4, PDF file, 0.02 MB. Copyright © 2023 Carter et al. 2023 Carter et al. https://creativecommons.org/licenses/by/4.0/ This content is distributed under the terms of the Creative Commons Attribution 4.0 International license.

10.1128/msystems.01003-22.7 FIG S5 Metagenome composition colored by the second-highest level of KO functional category and birth outcome of 72 PIN participants included in analysis, ordered by a whole-metagenome sequencing hierarchical clustering dendrogram. Shown is a dendrogram from hierarchical clustering of WMGS KO relative abundance data. The resulting WMGS clusters are indicated by the brackets to the right of each stacked bar plot. The taxon relative abundance from 16S rRNA gene amplicon sequencing data and KO functional category relative abundance from observed whole-metagenome sequencing data are presented. WMGS, whole-metagenome sequencing; KO, KEGG ortholog; KEGG, Kyoto Encyclopedia of Genes and Genomes. Download FIG S5, PDF file, 0.04 MB. Copyright © 2023 Carter et al. 2023 Carter et al. https://creativecommons.org/licenses/by/4.0/ This content is distributed under the terms of the Creative Commons Attribution 4.0 International license.

10.1128/msystems.01003-22.8 FIG S6 Vaginal metagenome α diversity. α diversity was estimated using WMGS KO relative abundance data. The α-diversity metric is indicated by the label at the top of each panel. “Observed” refers to the number of KOs observed in a sample or the observed KO richness. Results were stratified and colored by WMGS KO hierarchical cluster in panel A and stratified and colored by 16S rRNA gene amplicon sequencing cluster in panel B. Download FIG S6, PDF file, 0.1 MB. Copyright © 2023 Carter et al. 2023 Carter et al. https://creativecommons.org/licenses/by/4.0/ This content is distributed under the terms of the Creative Commons Attribution 4.0 International license.

10.1128/msystems.01003-22.9 FIG S7 Relationship between 16S rRNA gene amplicon sequencing α diversity and WMGS KO α diversity. Shown are scatterplots of 16S rRNA gene amplicon sequencing α diversity (*x* axis) versus WMGS KO α diversity (*y* axis). Results are colored by cluster from hierarchical clustering of 16S rRNA gene amplicon sequencing data and presenting 16S cluster-specific LOESS (locally estimated scatterplot smoothing) trend lines in panel A and colored by cluster from hierarchical clustering of WMGS cluster and presenting WMGS cluster-specific LOESS trend lines in panel B. The α-diversity metric is indicated by the label at the top of each panel. “Observed” refers to the number of features observed in a sample: ASVs for 16S rRNA gene amplicon sequencing and KOs for WMGS. WMGS, whole-metagenome sequencing; KO, KEGG ortholog; ASV, amplicon sequence variant; KEGG, Kyoto Encyclopedia of Genes and Genomes. Download FIG S7, PDF file, 0.06 MB. Copyright © 2023 Carter et al. 2023 Carter et al. https://creativecommons.org/licenses/by/4.0/ This content is distributed under the terms of the Creative Commons Attribution 4.0 International license.

10.1128/msystems.01003-22.10 TABLE S1 STORMS reporting checklist. STORMS, Strengthening the Organization and Reporting of Microbiome Studies; NA, not applicable; MIxS, Minimum Information about any (x) Sequence; STROBE, Strengthening the Reporting of Observational studies in Epidemiology; LC-MS/MS, liquid chromatography-tandem mass spectrometry; STREGA, Strengthening the Reporting of Genetic Association Studies; FDR, false-discovery rate; MAG, metagenome-assembled genome. (Adapted from Mirzayi et al., Nat Med 27:1885–1892, 2021, https://doi.org/10.1038/s41591-021-01552-x.). Download Table S1, PDF file, 0.2 MB. Copyright © 2023 Carter et al. 2023 Carter et al. https://creativecommons.org/licenses/by/4.0/ This content is distributed under the terms of the Creative Commons Attribution 4.0 International license.

10.1128/msystems.01003-22.11 TABLE S2 Comparison of whole-metagenome sequencing cluster membership and 16S rRNA gene amplicon sequencing cluster membership. Per footnote 1, WMGS clusters are based on KO relative abundance data. WMGS, whole-metagenome sequencing; KO, KEGG ortholog; KEGG, Kyoto Encyclopedia of Genes and Genomes. Download Table S2, PDF file, 0.01 MB. Copyright © 2023 Carter et al. 2023 Carter et al. https://creativecommons.org/licenses/by/4.0/ This content is distributed under the terms of the Creative Commons Attribution 4.0 International license.

## Supplementary Material

Reviewer comments

## References

[1] Langille MGI, Zaneveld J, Caporaso JG, McDonald D, Knights D, Reyes JA, Clemente JC, Burkepile DE, Vega Thurber RL, Knight R, Beiko RG, Huttenhower C. 2013. Predictive functional profiling of microbial communities using 16S rRNA marker gene sequences. Nat Biotechnol 31:814–821. doi:10.1038/nbt.2676. 23975157 PMC3819121

[2] Wemheuer F, Taylor JA, Daniel R, Johnston E, Meinicke P, Thomas T, Wemheuer B. 2020. Tax4Fun2: prediction of habitat-specific functional profiles and functional redundancy based on 16S rRNA gene sequences. Environ Microbiome 15:11. doi:10.1186/s40793-020-00358-7. 33902725 PMC8067651

[3] Douglas GM, Maffei VJ, Zaneveld JR, Yurgel SN, Brown JR, Taylor CM, Huttenhower C, Langille MGI. 2020. PICRUSt2 for prediction of metagenome functions. Nat Biotechnol 38:685–688. doi:10.1038/s41587-020-0548-6. 32483366 PMC7365738

[4] Aßhauer KP, Wemheuer B, Daniel R, Meinicke P. 2015. Tax4Fun: predicting functional profiles from metagenomic 16S rRNA data. Bioinformatics 31:2882–2884. doi:10.1093/bioinformatics/btv287. 25957349 PMC4547618

[5] Iwai S, Weinmaier T, Schmidt BL, Albertson DG, Poloso NJ, Dabbagh K, DeSantis TZ. 2016. Piphillin: improved prediction of metagenomic content by direct inference from human microbiomes. PLoS One 11:e0166104. doi:10.1371/journal.pone.0166104. 27820856 PMC5098786

[6] Sun S, Jones RB, Fodor AA. 2020. Inference-based accuracy of metagenome prediction tools varies across sample types and functional categories. Microbiome 8:46. doi:10.1186/s40168-020-00815-y. 32241293 PMC7118876

[7] Narayan NR, Weinmaier T, Laserna-Mendieta EJ, Claesson MJ, Shanahan F, Dabbagh K, Iwai S, DeSantis TZ. 2020. Piphillin predicts metagenomic composition and dynamics from DADA2-corrected 16S rDNA sequences. BMC Genomics 21:56–56. doi:10.1186/s12864-019-6427-1. 31952477 PMC6967091

[8] Ravel J, Gajer P, Abdo Z, Schneider GM, Koenig SS, McCulle SL, Karlebach S, Gorle R, Russell J, Tacket CO. 2011. Vaginal microbiome of reproductive-age women. Proc Natl Acad Sci USA 108:4680–4687. doi:10.1073/pnas.1002611107. 20534435 PMC3063603

[9] Seo S-S, Oh HY, Lee J-K, Kong J-S, Lee DO, Kim MK. 2016. Combined effect of diet and cervical microbiome on the risk of cervical intraepithelial neoplasia. Clin Nutr 35:1434–1441. doi:10.1016/j.clnu.2016.03.019. 27075319

[10] Tamarelle J, de Barbeyrac B, Le Hen I, Thiebaut A, Bebear C, Ravel J, Delarocque-Astagneau E. 2018. Vaginal microbiota composition and association with prevalent Chlamydia trachomatis infection: a cross-sectional study of young women attending a STI clinic in France. Sex Transm Infect 94:616–618. doi:10.1136/sextrans-2017-053346. 29358524

[11] Mehta SD, Nandi D, Agingu W, Green SJ, Bhaumik DK, Bailey RC, Otieno F. 2020. Vaginal and penile microbiome associations with HSV-2 in women and their male sex partners. J Infect Dis 226:644–654. doi:10.1093/infdis/jiaa529. PMC9441199 32822500

[12] Kumar M, Murugesan S, Singh P, Saadaoui M, Elhag DA, Terranegra A, Kabeer BSA, Marr AK, Kino T, Brummaier T, McGready R, Nosten F, Chaussabel D, Al Khodor S. 2021. Vaginal microbiota and cytokine levels predict preterm delivery in Asian women. Front Cell Infect Microbiol 11:639665–639665. doi:10.3389/fcimb.2021.639665. 33747983 PMC7969986

[13] Petrova MI, Lievens E, Malik S, Imholz N, Lebeer S. 2015. Lactobacillus species as biomarkers and agents that can promote various aspects of vaginal health. Front Physiol 6:81. doi:10.3389/fphys.2015.00081. 25859220 PMC4373506

[14] Das B, Nair GB. 2019. Homeostasis and dysbiosis of the gut microbiome in health and disease. J Biosci 44:117. doi:10.1007/s12038-019-9926-y. 31719226

[15] Hagerty SL, Hutchison KE, Lowry CA, Bryan AD. 2020. An empirically derived method for measuring human gut microbiome alpha diversity: demonstrated utility in predicting health-related outcomes among a human clinical sample. PLoS One 15:e0229204. doi:10.1371/journal.pone.0229204. 32119675 PMC7051054

[16] Austin MN, Rabe LK, Srinivasan S, Fredricks DN, Wiesenfeld HC, Hillier SL. 2015. Mageeibacillus indolicus gen. nov., sp. nov.: a novel bacterium isolated from the female genital tract. Anaerobe 32:37–42. doi:10.1016/j.anaerobe.2014.12.003. 25482717 PMC4385425

[17] Holm JB, France MT, Ma B, McComb E, Robinson CK, Mehta A, Tallon LJ, Brotman RM, Ravel J. 2020. Comparative metagenome-assembled genome analysis of “Candidatus Lachnocurva vaginae,” formerly known as bacterial vaginosis-associated bacterium-1 (BVAB1). Front Cell Infect Microbiol 10:117. doi:10.3389/fcimb.2020.00117. 32296647 PMC7136613

[18] Srinivasan S, Beamer MA, Fiedler TL, Austin MN, Sizova MV, Strenk SM, Agnew KJ, Gowda GAN, Raftery D, Epstein SS, Fredricks DN, Hillier SL. 2021. Megasphaera lornae sp. nov., Megasphaera hutchinsoni sp. nov., and Megasphaera vaginalis sp. nov.: novel bacteria isolated from the female genital tract. Int J Syst Evol Microbiol 71:e004702. doi:10.1099/ijsem.0.004702. PMC8161547 33616513

[19] Vaneechoutte M, Guschin A, Van Simaey L, Gansemans Y, Van Nieuwerburgh F, Cools P. 2019. Emended description of Gardnerella vaginalis and description of Gardnerella leopoldii sp. nov., Gardnerella piotii sp. nov. and Gardnerella swidsinskii sp. nov., with delineation of 13 genomic species within the genus Gardnerella. Int J Syst Evol Microbiol 69:679–687. doi:10.1099/ijsem.0.003200. 30648938

[20] Abdill RJ, Adamowicz EM, Blekhman R. 2022. Public human microbiome data are dominated by highly developed countries. PLoS Biol 20:e3001536. doi:10.1371/journal.pbio.3001536. 35167588 PMC8846514

[21] Savitz DA, Dole N, Williams J, Thorp JM, McDonald T, Carter AC, Eucker B. 1999. Determinants of participation in an epidemiological study of preterm delivery. Paediatr Perinat Epidemiol 13:114–125. doi:10.1046/j.1365-3016.1999.00156.x. 9987790

[22] France MT, Mendes-Soares H, Forney LJ. 2016. Genomic comparisons of Lactobacillus crispatus and Lactobacillus iners reveal potential ecological drivers of community composition in the vagina. Appl Environ Microbiol 82:7063–7073. doi:10.1128/AEM.02385-16. 27694231 PMC5118917

[23] Ma B, France MT, Crabtree J, Holm JB, Humphrys MS, Brotman RM, Ravel J. 2020. A comprehensive non-redundant gene catalog reveals extensive within-community intraspecies diversity in the human vagina. Nat Commun 11:940. doi:10.1038/s41467-020-14677-3. 32103005 PMC7044274

[24] Macklaim JM, Gloor GB, Anukam KC, Cribby S, Reid G. 2011. At the crossroads of vaginal health and disease, the genome sequence of Lactobacillus iners AB-1. Proc Natl Acad Sci USA 108:4688–4695. doi:10.1073/pnas.1000086107. 21059957 PMC3063587

[25] Mendes-Soares H, Suzuki H, Hickey RJ, Forney LJ. 2014. Comparative functional genomics of Lactobacillus spp. reveals possible mechanisms for specialization of vaginal lactobacilli to their environment. J Bacteriol 196:1458–1470. doi:10.1128/JB.01439-13. 24488312 PMC3993339

[26] Franzosa EA, McIver LJ, Rahnavard G, Thompson LR, Schirmer M, Weingart G, Lipson KS, Knight R, Caporaso JG, Segata N, Huttenhower C. 2018. Species-level functional profiling of metagenomes and metatranscriptomes. Nat Methods 15:962–968. doi:10.1038/s41592-018-0176-y. 30377376 PMC6235447

[27] Chen Q, Galfalvy H, Duan N. 2013. Effects of disease misclassification on exposure-disease association. Am J Public Health 103:e67–e73. doi:10.2105/AJPH.2012.300995. PMC3698812 23488509

[28] Jurek AM, Greenland S, Maldonado G. 2008. How far from non-differential does exposure or disease misclassification have to be to bias measures of association away from the null? Int J Epidemiol 37:382–385. doi:10.1093/ije/dym291. 18184671

[29] Gosmann C, Anahtar MN, Handley SA, Farcasanu M, Abu-Ali G, Bowman BA, Padavattan N, Desai C, Droit L, Moodley A, Dong M, Chen Y, Ismail N, Ndung'u T, Ghebremichael MS, Wesemann DR, Mitchell C, Dong KL, Huttenhower C, Walker BD, Virgin HW, Kwon DS. 2017. Lactobacillus-deficient cervicovaginal bacterial communities are associated with increased HIV acquisition in young South African women. Immunity 46:29–37. doi:10.1016/j.immuni.2016.12.013. 28087240 PMC5270628

[30] France MT, Ma B, Gajer P, Brown S, Humphrys MS, Holm JB, Waetjen LE, Brotman RM, Ravel J. 2020. VALENCIA: a nearest centroid classification method for vaginal microbial communities based on composition. Microbiome 8:166. doi:10.1186/s40168-020-00934-6. 33228810 PMC7684964

[31] Carter KA, Fischer MD, Petrova MI, Balkus JE. 2022. Epidemiologic evidence on the role of Lactobacillus iners in sexually transmitted infections and bacterial vaginosis: a series of systematic reviews and meta-analyses. Sex Transm Dis doi:10.1097/OLQ.0000000000001744. PMC10006306 36729966

[32] MacIntyre DA, Chandiramani M, Lee YS, Kindinger L, Smith A, Angelopoulos N, Lehne B, Arulkumaran S, Brown R, Teoh TG, Holmes E, Nicoholson JK, Marchesi JR, Bennett PR. 2015. The vaginal microbiome during pregnancy and the postpartum period in a European population. Sci Rep 5:8988. doi:10.1038/srep08988. 25758319 PMC4355684

[33] Aagaard K, Riehle K, Ma J, Segata N, Mistretta T-A, Coarfa C, Raza S, Rosenbaum S, Van den Veyver I, Milosavljevic A, Gevers D, Huttenhower C, Petrosino J, Versalovic J. 2012. A metagenomic approach to characterization of the vaginal microbiome signature in pregnancy. PLoS One 7:e36466. doi:10.1371/journal.pone.0036466. 22719832 PMC3374618

[34] Freitas AC, Chaban B, Bocking A, Rocco M, Yang S, Hill JE, Money DM, Hemmingsen S, Reid G, Dumonceaux T, Gloor G, Links M, O’Doherty K, Tang P, van Schalkwyk J, Yudin M, The VOGUE Research Group. 2017. The vaginal microbiome of pregnant women is less rich and diverse, with lower prevalence of Mollicutes, compared to non-pregnant women. Sci Rep 7:9212. doi:10.1038/s41598-017-07790-9. 28835692 PMC5569030

[35] Nunn KL, Witkin SS, Schneider GM, Boester A, Nasioudis D, Minis E, Gliniewicz K, Forney LJ. 2021. Changes in the vaginal microbiome during the pregnancy to postpartum transition. Reprod Sci 28:1996–2005. doi:10.1007/s43032-020-00438-6. 33432532 PMC8189965

[36] Carter KA, Balkus JE, Anzala O, Kimani J, Hoffman NG, Fiedler TL, Mochache V, Fredricks DN, McClelland RS, Srinivasan S. 2022. Associations between vaginal bacteria and bacterial vaginosis signs and symptoms: a comparative study of Kenyan and American women. Front Cell Infect Microbiol 12:801770. doi:10.3389/fcimb.2022.801770. 35310847 PMC8931342

[37] Jin L, Tao L, Pavlova S, So J, Kiwanuka N, Namukwaya Z, Saberbein B, Wawer M. 2007. Species diversity and relative abundance of vaginal lactic acid bacteria from women in Uganda and Korea. J Appl Microbiol 102:1107–1115. doi:10.1111/j.1365-2672.2006.03147.x. 17381754

[38] Benning L, Golub ET, Anastos K, French AL, Cohen M, Gilbert D, Gillevet P, Munyazesa E, Landay AL, Sikaroodi M, Spear GT. 2014. Comparison of lower genital tract microbiota in HIV-infected and uninfected women from Rwanda and the US. PLoS One 9:e96844. doi:10.1371/journal.pone.0096844. 24817204 PMC4016010

[39] Borgdorff H, van der Veer C, van Houdt R, Alberts CJ, de Vries HJ, Bruisten SM, Snijder MB, Prins M, Geerlings SE, Schim van der Loeff MF, van de Wijgert JHHM. 2017. The association between ethnicity and vaginal microbiota composition in Amsterdam, the Netherlands. PLoS One 12:e0181135. doi:10.1371/journal.pone.0181135. 28700747 PMC5507447

[40] Marconi C, El-Zein M, Ravel J, Ma B, Lima MD, Carvalho NS, Alves RRF, Parada CMGL, Leite SHM, Giraldo PC, Gonçalves AK, Franco EL, Silva MG. 2020. Characterization of the vaginal microbiome in women of reproductive age from 5 regions in Brazil. Sex Transm Dis 47:562–569. doi:10.1097/OLQ.0000000000001204. 32520883

[41] Mirzayi C, Renson A, Genomic Standards Consortium, Molecular Analysis and Quality Control Society, Furlanello C, Sansone S-A, Zohra F, Elsafoury S, Geistlinger L, Kasselman LJ, Eckenrode K, van de Wijgert J, Loughman A, Marques FZ, MacIntyre DA, Arumugam M, Azhar R, Beghini F, Bergstrom K, Bhatt A, Bisanz JE, Braun J, Bravo HC, Buck GA, Bushman F, Casero D, Clarke G, Collado MC, Cotter PD, Cryan JF, Demmer RT, Devkota S, Elinav E, Escobar JS, Fettweis J, Finn RD, Fodor AA, Forslund S, Franke A, Furlanello C, Gilbert J, Grice E, Haibe-Kains B, Handley S, Herd P, Holmes S, Jacobs JP, Karstens L, Knight R, Knights D, Koren O, Kwon DS, et al. 2021. Reporting guidelines for human microbiome research: the STORMS checklist. Nat Med 27:1885–1892. doi:10.1038/s41591-021-01552-x. 34789871 PMC9105086

[42] Nugent RP, Krohn MA, Hillier SL. 1991. Reliability of diagnosing bacterial vaginosis is improved by a standardized method of Gram stain interpretation. J Clin Microbiol 29:297–301. doi:10.1128/jcm.29.2.297-301.1991. 1706728 PMC269757

[43] Sun S, Serrano MG, Fettweis JM, Basta P, Rosen E, Ludwig K, Sorgen AA, Blakley IC, Wu MC, Dole N, Thorp JM, Siega-Riz AM, Buck GA, Fodor AA, Engel SM. 2022. Race, the vaginal microbiome, and spontaneous preterm birth. mSystems 7:e00017-22. doi:10.1128/msystems.00017-22. 35582911 PMC9238383

[44] Fettweis JM, Serrano MG, Brooks JP, Edwards DJ, Girerd PH, Parikh HI, Huang B, Arodz TJ, Edupuganti L, Glascock AL, Xu J, Jimenez NR, Vivadelli SC, Fong SS, Sheth NU, Jean S, Lee V, Bokhari YA, Lara AM, Mistry SD, Duckworth RA, Bradley SP, Koparde VN, Orenda XV, Milton SH, Rozycki SK, Matveyev AV, Wright ML, Huzurbazar SV, Jackson EM, Smirnova E, Korlach J, Tsai Y-C, Dickinson MR, Brooks JL, Drake JI, Chaffin DO, Sexton AL, Gravett MG, Rubens CE, Wijesooriya NR, Hendricks-Muñoz KD, Jefferson KK, Strauss JF, Buck GA. 2019. The vaginal microbiome and preterm birth. Nat Med 25:1012–1021. doi:10.1038/s41591-019-0450-2. 31142849 PMC6750801

[45] Serrano MG, Parikh HI, Brooks JP, Edwards DJ, Arodz TJ, Edupuganti L, Huang B, Girerd PH, Bokhari YA, Bradley SP, Brooks JL, Dickinson MR, Drake JI, Duckworth RA, Fong SS, Glascock AL, Jean S, Jimenez NR, Khoury J, Koparde VN, Lara AM, Lee V, Matveyev AV, Milton SH, Mistry SD, Rozycki SK, Sheth NU, Smirnova E, Vivadelli SC, Wijesooriya NR, Xu J, Xu P, Chaffin DO, Sexton AL, Gravett MG, Rubens CE, Hendricks-Muñoz KD, Jefferson KK, Strauss JF, Fettweis JM, Buck GA. 2019. Racioethnic diversity in the dynamics of the vaginal microbiome during pregnancy. Nat Med 25:1001–1011. doi:10.1038/s41591-019-0465-8. 31142850 PMC6746180

[46] Fettweis JM, Brooks JP, Serrano MG, Sheth NU, Girerd PH, Edwards DJ, Strauss JF, The Vaginal Microbiome Consortium, Jefferson KK, Buck GA. 2014. Differences in vaginal microbiome in African American women versus women of European ancestry. Microbiology (Reading) 160:2272–2282. doi:10.1099/mic.0.081034-0. 25073854 PMC4178329

[47] Jean S, Huang B, Parikh HI, Edwards DJ, Brooks JP, Kumar NG, Sheth NU, Koparde V, Smirnova E, Huzurbazar S, Girerd PH, Wijesinghe DS, Strauss JFI, Serrano MG, Fettweis JM, Jefferson KK, Buck GA. 2019. Multi-omic microbiome profiles in the female reproductive tract in early pregnancy. Infect Microbes Dis 1:49–60. doi:10.1097/IM9.0000000000000007.

[48] Bolyen E, Rideout JR, Dillon MR, Bokulich NA, Abnet CC, Al-Ghalith GA, Alexander H, Alm EJ, Arumugam M, Asnicar F, Bai Y, Bisanz JE, Bittinger K, Brejnrod A, Brislawn CJ, Brown CT, Callahan BJ, Caraballo-Rodríguez AM, Chase J, Cope EK, Da Silva R, Diener C, Dorrestein PC, Douglas GM, Durall DM, Duvallet C, Edwardson CF, Ernst M, Estaki M, Fouquier J, Gauglitz JM, Gibbons SM, Gibson DL, Gonzalez A, Gorlick K, Guo J, Hillmann B, Holmes S, Holste H, Huttenhower C, Huttley GA, Janssen S, Jarmusch AK, Jiang L, Kaehler BD, Kang KB, Keefe CR, Keim P, Kelley ST, Knights D, et al. 2019. Reproducible, interactive, scalable and extensible microbiome data science using QIIME 2. Nat Biotechnol 37:852–857. doi:10.1038/s41587-019-0209-9. 31341288 PMC7015180

[49] Callahan BJ, McMurdie PJ, Rosen MJ, Han AW, Johnson AJA, Holmes SP. 2016. DADA2: high-resolution sample inference from Illumina amplicon data. Nat Methods 13:581–583. doi:10.1038/nmeth.3869. 27214047 PMC4927377

[50] Quast C, Pruesse E, Yilmaz P, Gerken J, Schweer T, Yarza P, Peplies J, Glöckner FO. 2013. The SILVA ribosomal RNA gene database project: improved data processing and web-based tools. Nucleic Acids Res 41:D590–D596. doi:10.1093/nar/gks1219. 23193283 PMC3531112

[51] Glöckner FO, Yilmaz P, Quast C, Gerken J, Beccati A, Ciuprina A, Bruns G, Yarza P, Peplies J, Westram R, Ludwig W. 2017. 25 years of serving the community with ribosomal RNA gene reference databases and tools. J Biotechnol 261:169–176. doi:10.1016/j.jbiotec.2017.06.1198. 28648396

[52] Yilmaz P, Parfrey LW, Yarza P, Gerken J, Pruesse E, Quast C, Schweer T, Peplies J, Ludwig W, Glöckner FO. 2014. The SILVA and “All-species Living Tree Project (LTP)” taxonomic frameworks. Nucleic Acids Res 42:D643–D648. doi:10.1093/nar/gkt1209. 24293649 PMC3965112

[53] Oskooi AF, Roundy D, Ibanescu M, Bermel P, Joannopoulos JD, Johnson SG. 2010. MEEP: a flexible free-software package for electromagnetic simulations by the FDTD method. Comput Phys Commun 181:687–702. doi:10.1016/j.cpc.2009.11.008.

[54] Koparde VN, Parikh HI, Bradley SP, Sheth NU. 2015. MEEPTOOLS: a maximum expected error based FASTQ read filtering and trimming toolkit. arXiv 151203344. https://arxiv.org/ftp/arxiv/papers/1512/1512.03344.pdf.

[55] Li H, Durbin R. 2009. Fast and accurate short read alignment with Burrows-Wheeler transform. Bioinformatics 25:1754–1760. doi:10.1093/bioinformatics/btp324. 19451168 PMC2705234

[56] Martin J, Sykes S, Young S, Kota K, Sanka R, Sheth N, Orvis J, Sodergren E, Wang Z, Weinstock GM, Mitreva M. 2012. Optimizing read mapping to reference genomes to determine composition and species prevalence in microbial communities. PLoS One 7:e36427. doi:10.1371/journal.pone.0036427. 22719831 PMC3374613

[57] Kanehisa M, Goto S. 2000. KEGG: Kyoto Encyclopedia of Genes and Genomes. Nucleic Acids Res 28:27–30. doi:10.1093/nar/28.1.27. 10592173 PMC102409

[58] Kanehisa M. 2019. Toward understanding the origin and evolution of cellular organisms. Protein Sci 28:1947–1951. doi:10.1002/pro.3715. 31441146 PMC6798127

[59] Kanehisa M, Furumichi M, Sato Y, Ishiguro-Watanabe M, Tanabe M. 2021. KEGG: integrating viruses and cellular organisms. Nucleic Acids Res 49:D545–D551. doi:10.1093/nar/gkaa970. 33125081 PMC7779016

