# Supplementary figures and images for: Vaginal Microbiome Metagenome Inference Accuracy: Differential Measurement Error according to Community Composition
Source: mSystems. 2023 Mar 28;8(2):e01003-22. doi: 10.1128/msystems.01003-22 (PMC10134888; doi:10.1128/msystems.01003-22)

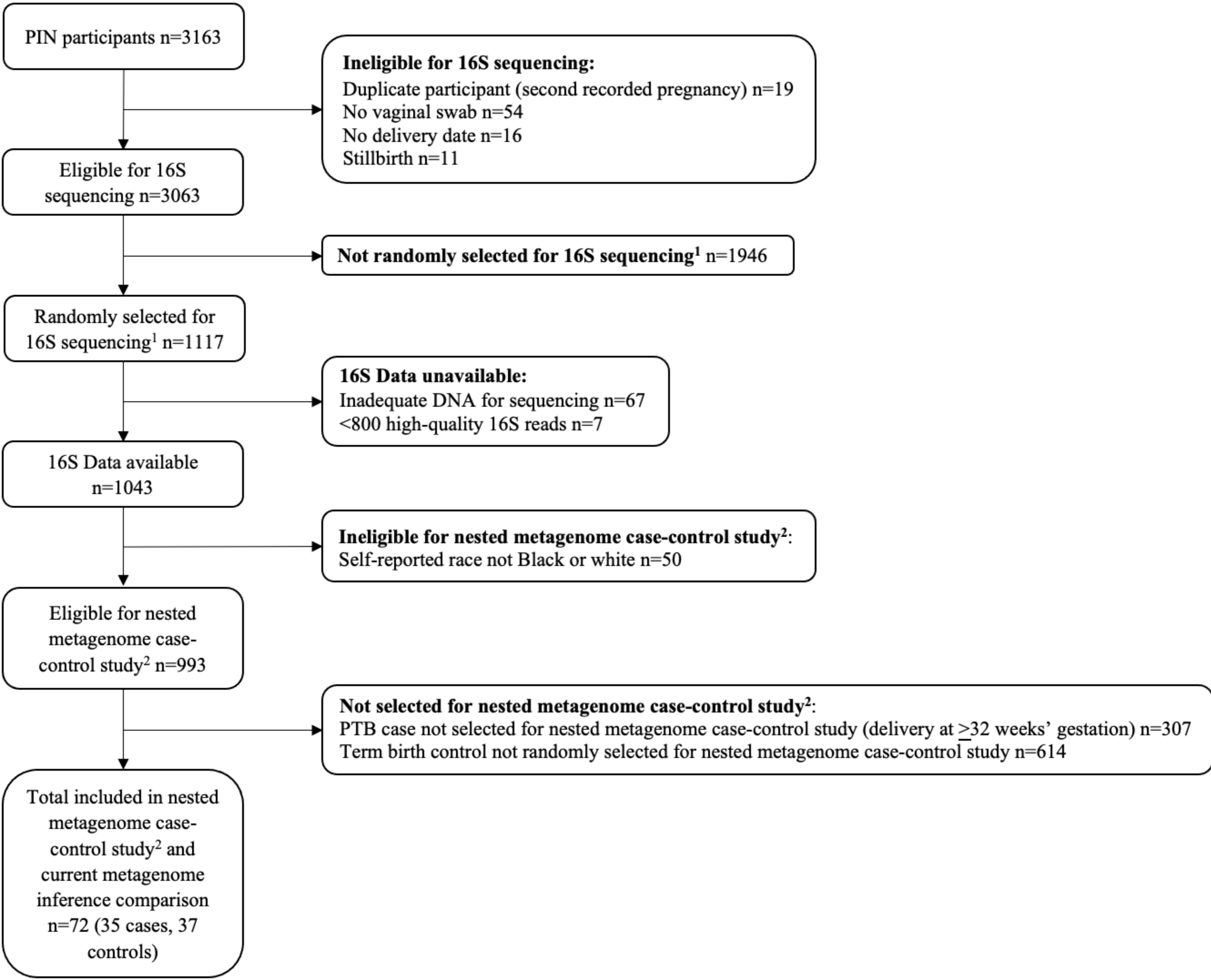

Supplement: FIG S1 [file msystems.01003-22-s0002.pdf]

A

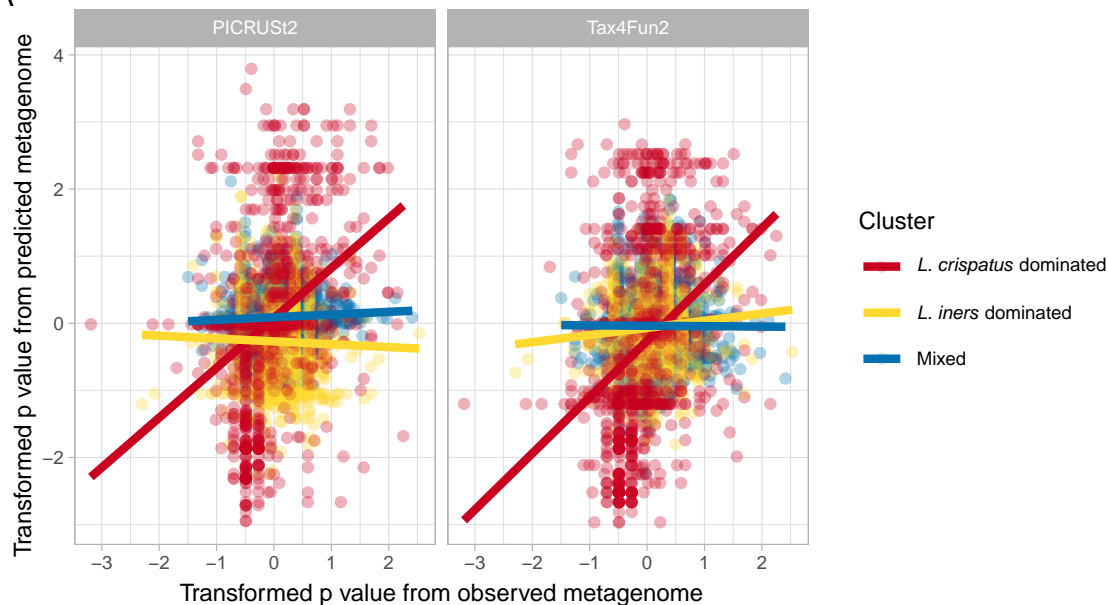

B

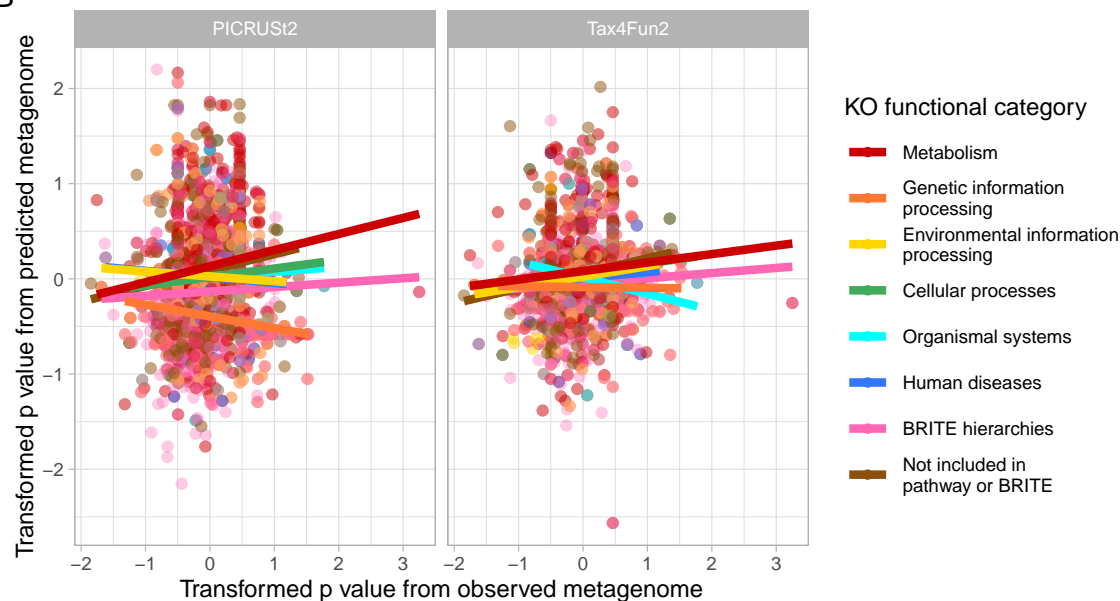

Supplement: FIG S3 [file msystems.01003-22-s0006.pdf]

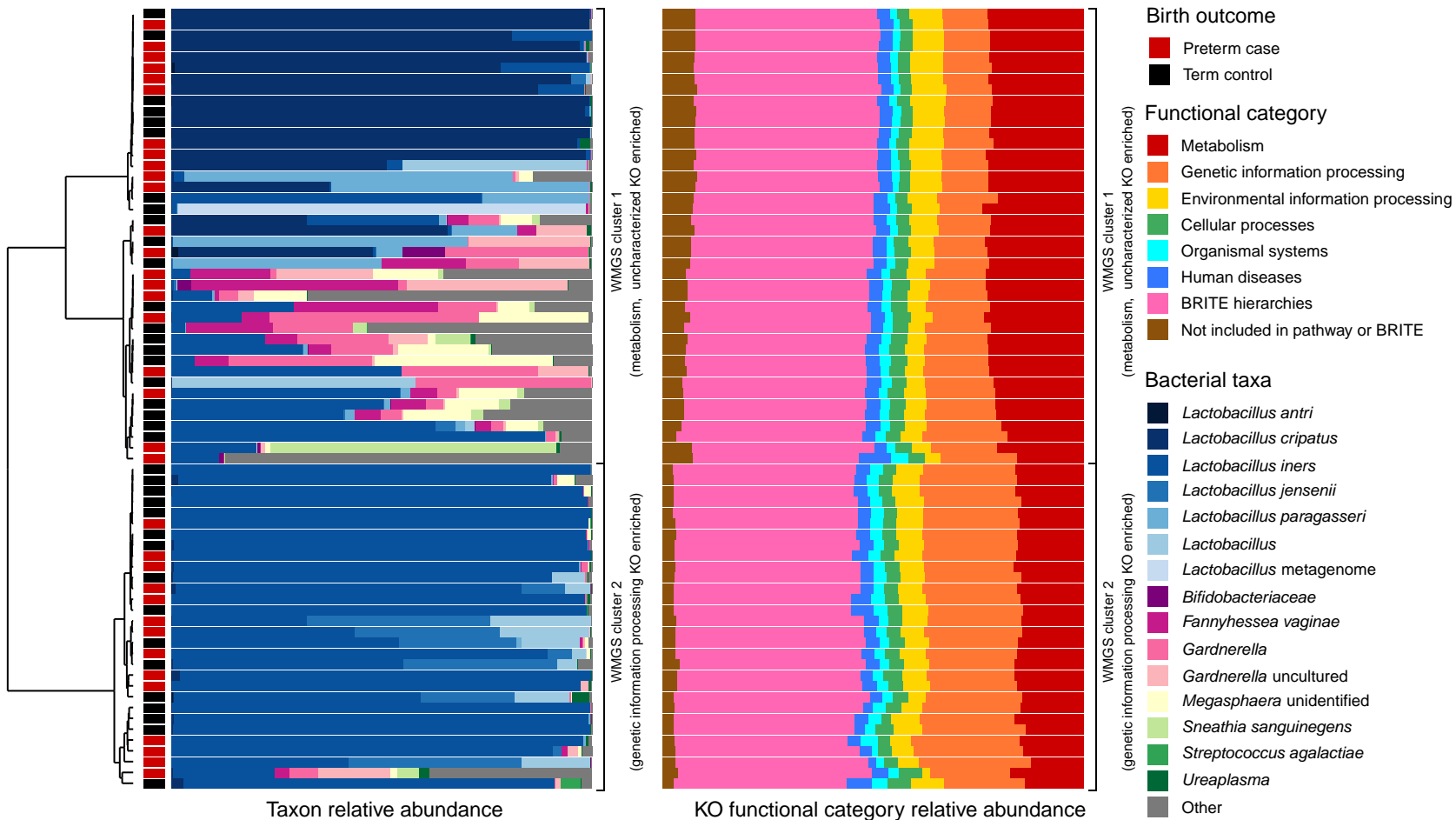

Supplement: FIG S4 [file msystems.01003-22-s0007.pdf]

A

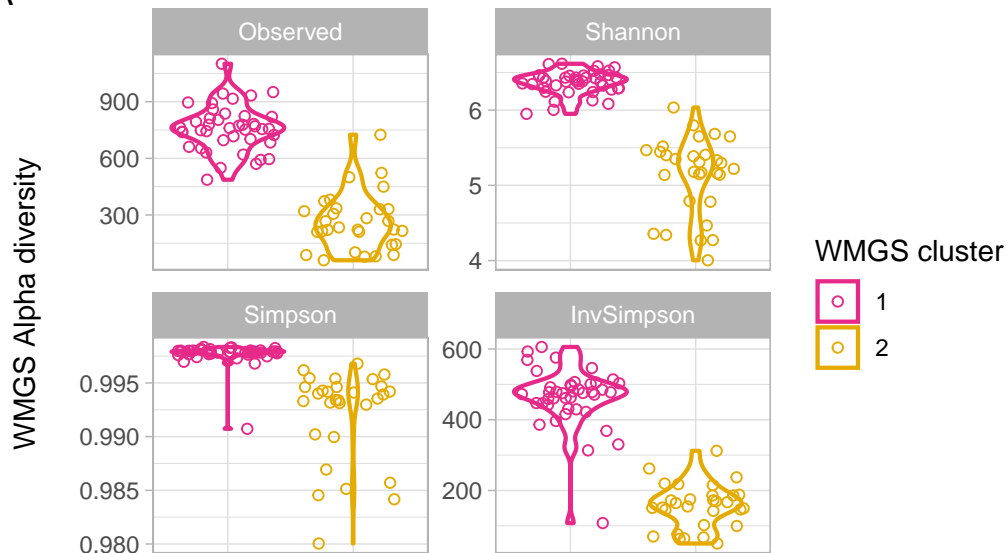

B

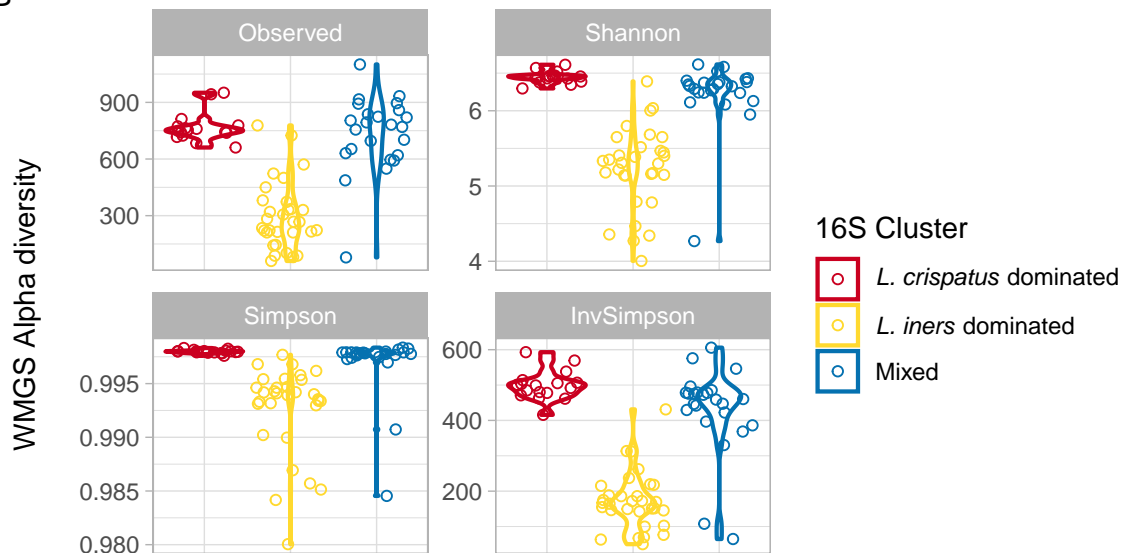

Supplement: FIG S6 [file msystems.01003-22-s0009.pdf]

A

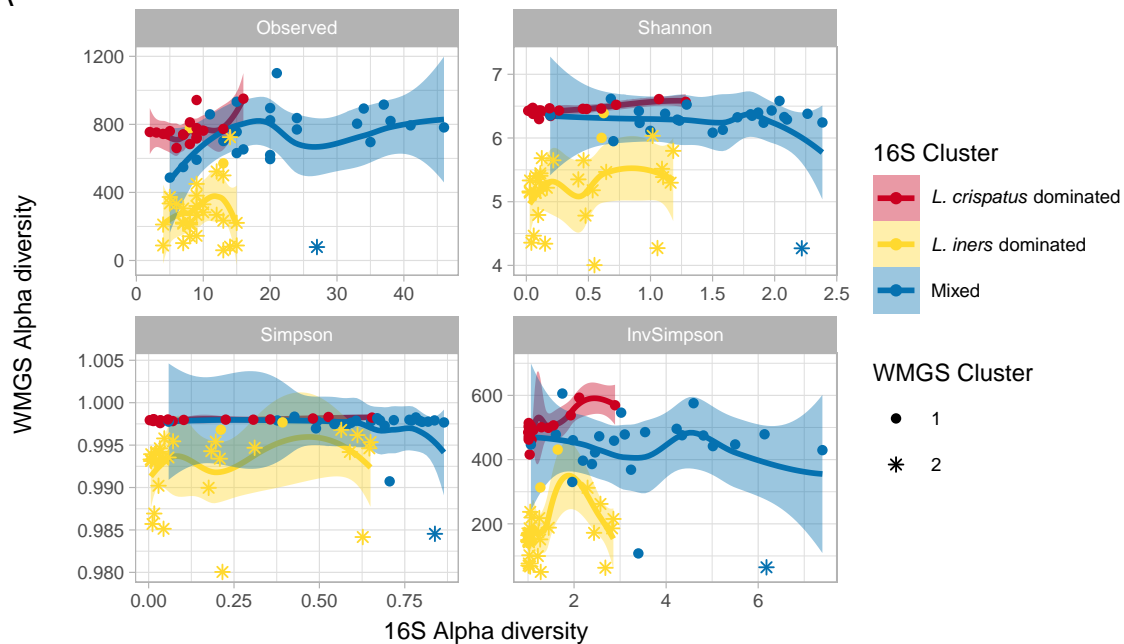

B

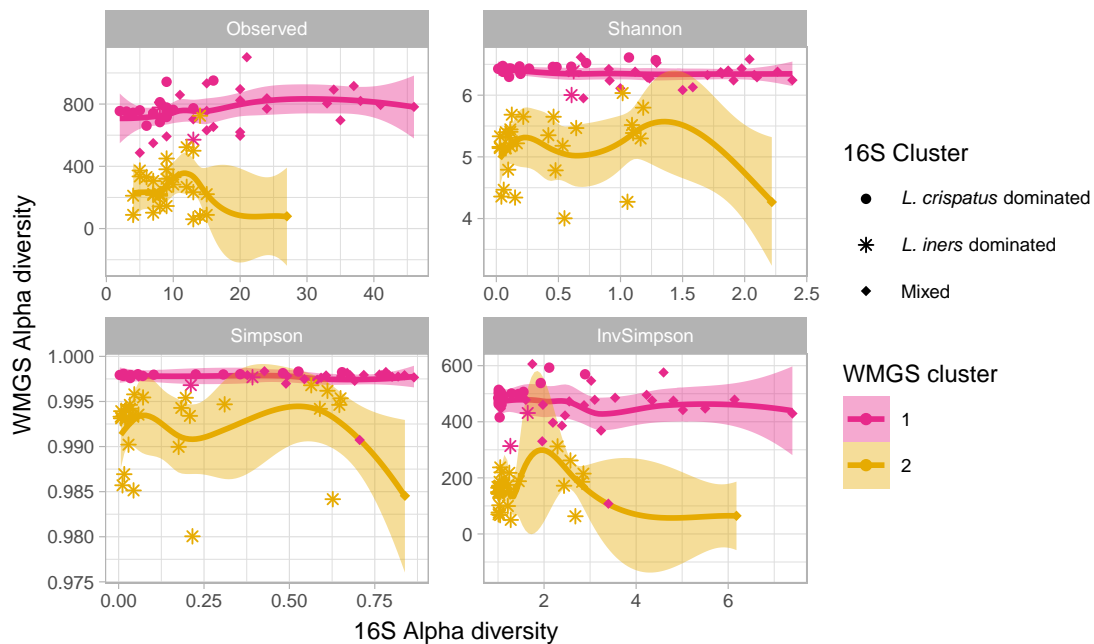

Supplement: FIG S7 [file msystems.01003-22-s0010.pdf]
