# Supplementary material for: Vaginal Microbiome Metagenome Inference Accuracy: Differential Measurement Error according to Community Composition
Source: mSystems. 2023 Mar 28;8(2):e01003-22. doi: 10.1128/msystems.01003-22 (PMC10134888; doi:10.1128/msystems.01003-22)
Supplement: TEXT S1 [file msystems.01003-22-s0004.pdf]

## Summary of hierarchical clusters and alpha diversity estimates generated from whole metagenome sequencing data

We performed hierarchical clustering of whole metagenome sequencing (WMGS) data using KEGG ortholog (KO) relative abundances. According to all three internal validation statistics (connectivity, Dunn, silhouette), the optimal number of clusters was two. Forty-two samples belonged to WMGS cluster 1 (58% of total), including 21 preterm birth (PTB) cases (60% of cases), and 30 samples belonged to WMGS cluster 2 (42% of total), including 14 PTB cases (40% of cases, Figures S4, S5). Compared to WMGS cluster 2, WMGS cluster 1 was marked by enrichment of KOs involved in metabolism and uncharacterized KOs. Compared to WMGS cluster 1, WMGS cluster 2 was marked by enrichment of genetic information processing KOs.

The ranges of WMGS KO alpha diversity values overlapped between WMGS clusters (Figure S6). However, samples belonging to WMGS cluster 1 (metabolism, uncharacterized KO enriched) tended to have higher alpha diversity than samples belonging to WMGS cluster 2 (genetic information processing KO enriched).

## Comparison of WMGS and 16S rRNA gene amplicon sequencing clusters

Cluster membership based on 16S rRNA gene amplicon sequencing and WMGS data is cross-tabulated in Table S2. WMGS cluster 1 (metabolism, uncharacterized KO enriched) included all *L. crispatus*-dominated samples, all but one mixed sample, and two *L. iners*-dominated samples. WMGS cluster 2 (genetic information processing KO enriched)

included all remaining *L. iners*-dominated samples (n = 29, 94%) and the remaining mixed sample.

Within 16S rRNA gene amplicon sequencing clusters, WMGS KO alpha diversity was largely overlapping between *L. crispatus*-dominated and mixed samples, which tended to show higher KO alpha diversity than *L. iners*-dominated samples (Figure S6). *L. crispatus*-dominated samples showed the narrowest range and most right-shifted distribution (highest values) of KO alpha diversity, which is in contrast with alpha diversity estimated from 16S rRNA gene amplicon sequencing both in our data and as has been observed in the field more broadly.

Figure S7 display the relationship between alpha diversity estimated from 16S rRNA gene amplicon sequencing and WMGS data. On the whole, there appear to be null-to-positive relationships between 16S rRNA gene amplicon sequencing and WMGS alpha diversity, depending on the diversity metric considered. Within 16S rRNA gene amplicon sequencing and WMGS clusters, no clear or consistent trends emerge, with several trend lines being nonmonotonic or approximately flat.

Considering these data, we elected not to run an additional analysis comparing metagenome inference performance stratified by WMGS cluster. Because WMGS cluster 1 was almost exclusively comprised of *L. crispatus*-dominated and mixed samples while WMGS cluster 2 was almost exclusively comprised of *L. iners*-dominated samples, and because differential metagenome inference performance in our analysis was driven by

differences in metagenome content between *L. crispatus*- and *L. iners*-dominated samples and underrepresentation of *L. iners* in reference sequence databases, we expect PICRUSt2 and Tax4Fun2 to perform better for WMGS cluster 1 than for WMGS cluster 2. Because this is a reasonable assumption and because WMGS clusters are less readily interpretable than 16S rRNA gene amplicon sequencing clusters, an additional analysis stratified by WMGS cluster would add little additional value to the analysis.
