# Supplementary material for: Vaginal Microbiome Metagenome Inference Accuracy: Differential Measurement Error according to Community Composition
Source: mSystems. 2023 Mar 28;8(2):e01003-22. doi: 10.1128/msystems.01003-22 (PMC10134888; doi:10.1128/msystems.01003-22)
Supplement: FIG S5 [file msystems.01003-22-s0008.pdf]

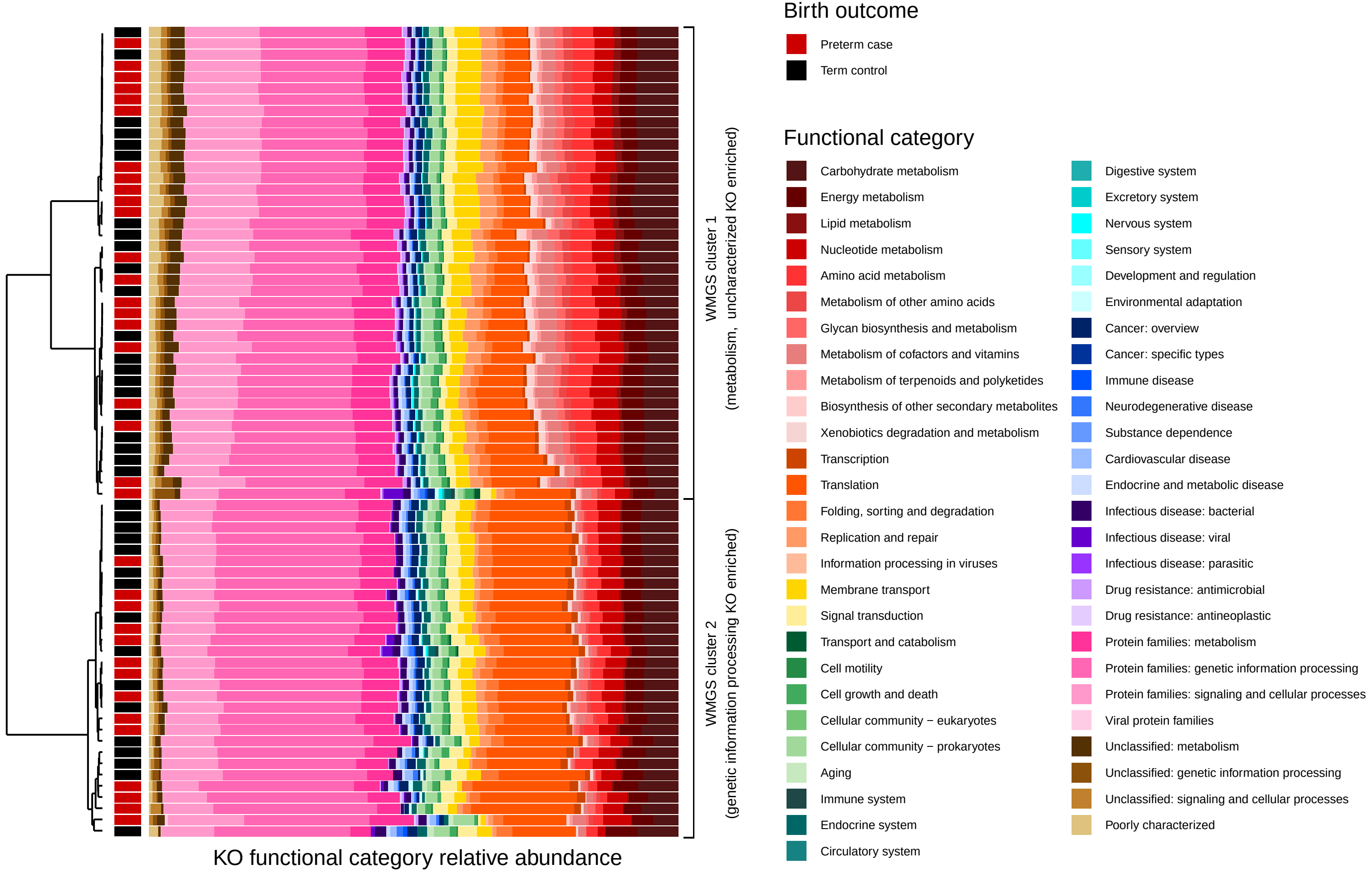
Birth outcome
Preterm case
Term control
Functional category
Carbohydrate metabolism
Energy metabolism
Lipid metabolism
Nucleotide metabolism
Amino acid metabolism
Metabolism of other amino acids
Glycan biosynthesis and metabolism
Metabolism of cofactors and vitamins
Metabolism of terpenoids and polyketides
Biosynthesis of other secondary metabolites
Xenobiotics degradation and metabolism
Transcription
Translation
Folding, sorting and degradation
Replication and repair
Information processing in viruses
Membrane transport
Signal transduction
Transport and catabolism
Cell motility
Cell growth and death
Cellular community − eukaryotes
Cellular community − prokaryotes
Aging
Immune system
Endocrine system
Circulatory system
Digestive system
Excretory system
Nervous system
Sensory system
Development and regulation
Environmental adaptation
Cancer: overview
Cancer: specific types
Immune disease
Neurodegenerative disease
Substance dependence
Cardiovascular disease
Endocrine and metabolic disease
Infectious disease: bacterial
Infectious disease: viral
Infectious disease: parasitic
Drug resistance: antimicrobial
Drug resistance: antineoplastic
Protein families: metabolism
Protein families: genetic information processing
Protein families: signaling and cellular processes
Viral protein families
Unclassified: metabolism
Unclassified: genetic information processing
Unclassified: signaling and cellular processes
Poorly characterized
WMGS cluster 1
(metabolism, uncharacterized KO enriched)
WMGS cluster 2
(genetic information processing KO enriched)
KO functional category relative abundance
