# Supplementary material for: Vaginal Microbiome Metagenome Inference Accuracy: Differential Measurement Error according to Community Composition
Source: mSystems. 2023 Mar 28;8(2):e01003-22. doi: 10.1128/msystems.01003-22 (PMC10134888; doi:10.1128/msystems.01003-22)
Supplement: TABLE S2 [file msystems.01003-22-s0005.pdf]

16S rRNA gene amplicon sequencing cluster	WMGS cluster[1]			
	Cluster 1 (metabolism, uncharacterized KO enriched) (N = 42)		Cluster 2 (genetic information processing KO enriched) (N = 30)	
	n	%	n	%
L. crispatus-dominated	17	40	0	0
L. iners-dominated	2	5	29	97
Mixed	23	55	1	3
